# Functional E3 ligase hotspots and resistance mechanisms to small-molecule degraders

**DOI:** 10.1038/s41589-022-01177-2

**Published:** 2022-11-03

**Authors:** Alexander Hanzl, Ryan Casement, Hana Imrichova, Scott J. Hughes, Eleonora Barone, Andrea Testa, Sophie Bauer, Jane Wright, Matthias Brand, Alessio Ciulli, Georg E. Winter

**Affiliations:** 1 CeMM Research Center for Molecular Medicine of the Austrian Academy of Sciences, 1090 Vienna, Austria; 2 Division of Biological Chemistry and Drug Discovery, School of Life Sciences, University of Dundee, James Black Centre, Dundee, UK

## Abstract

Targeted protein degradation is a novel pharmacology established by drugs that recruit target proteins to E3 ubiquitin ligases. Based on the structure of the degrader and the target, different E3 interfaces are critically involved, thus forming defined “functional hotspots”. Understanding disruptive mutations in functional hotspots informs on the architecture of the assembly, and highlights residues susceptible to acquire resistance phenotypes. Here, we employ haploid genetics to show that hotspot mutations cluster in substrate receptors of hijacked ligases, where mutation type and frequency correlate with gene essentiality. Intersection with deep mutational scanning revealed hotspots that are conserved or specific for chemically distinct degraders and targets. Biophysical and structural validation suggests that hotspot mutations frequently converge on altered ternary complex assembly. Moreover, we validated hotspots mutated in patients that relapse from degrader treatment. In sum, we present a fast and widely accessible methodology to characterize small-molecule degraders and associated resistance mechanisms.

## Introduction

Proximity-inducing pharmacology is a therapeutic paradigm of current great interest in academia and industry^1^. It is based on small molecules that co-opt the function of one protein by inducing a naturally non-occurring or non-consequential interaction with another protein^2^. One of the most powerful embodiments of proximity-inducing pharmacology is the concept of targeted protein degradation (TPD). In TPD, small-molecule “degraders” induce the molecular proximity between an E3 ubiquitin ligase and a protein of interest (POI), leading to the poly-ubiquitination and proteasomal degradation of the POI^3^. Degraders are typically categorized either as heterobifunctional PROTACs, or as monovalent molecular glues. Many of the E3 ligases that are currently amenable to TPD are members of the large family of cullin RING E3 ubiquitin ligases (CRL)^4–6^. CRLs are modular protein assemblies that are organized around a central cullin backbone. This also includes the two ligases most commonly hijacked by degraders that have reached clinical evaluation or approval, namely CRL2^VHL^ and CRL4^CRBN^ ^7^. The specificity of substrate recognition among CRLs is conveyed by more than 250 different substrate receptors (SR), such as the aforementioned cereblon (CRBN) and von Hippel-Lindau disease tumor suppressor (VHL). In physiological settings, SRs recognize substrates for instance based on posttranslational modifications. The underpinning molecular recognition is hence based on complementary and co-evolved protein surfaces. Based on the natural, highly diversified function of SRs, they are ideal entry points for small-molecule modulation.

While naturally occurring substrate recognition is evolutionary optimized, small-molecule degraders often induce the formation of *de novo* protein-protein interactions^2,8,9^. As a result, degraders rely on an optimal exploitation of the structural plasticity of both involved protein surfaces and leveraging PPI energetics from the induced proximity. Successfully designed degraders induce a tripartite binding between SR, degrader, and POI, which is correctly positioned and sufficiently stable to ensure effective poly-ubiquitination and degradation of the POI. While cooperativity of the ternary complex formation is not required, it is often positively correlated with degrader potency^10–12^. Hence, variations in the geometry and PPIs of the states reflecting the drug-induced ternary complex ensemble may give rise to different “functional hotspots” in the hijacked ligase. We define functional hotspots as the repertoire of amino acid residues that affect drug potency upon substitution. Identification of such hotspots would allow prediction of putative mechanisms of degrader resistance. This could consequently further advance our understanding of cellular determinants of degrader efficacy^13–16^. Inspired by advances in the field of overcoming kinase inhibitor resistance^17^, we anticipate that a detailed map of functional SR hotspots could inform on strategies to optimize degrader design to overcome or even prevent resistance acquisition.

Currently, identification of functional hotspots is predominantly driven by structural biology. Structural elucidation has been instrumental in shaping our understanding of TPD, and also empowers predictive computational models of ternary complex assembly^18–21^. However, it also faces some crucial limitations. Among others, structures (i) present a static snapshot of an otherwise dynamic system, (ii) might lack resolution especially at dynamic interfaces, (iii) don’t consider stoichiometry found in a cellular environment and (iv) often depend on truncated protein constituents lacking posttranslational modifications. Complementary in solution technologies, such as Hydrogen Deuterium Exchange Mass Spectrometry (HDX-MS) and small-angle X-ray scattering, can provide a more dynamic perspective, even though many of the aforementioned aspects and limitations similarly apply^22,23^.

Here we set out to bridge this gap by integrating genomics approaches that enable an *in cellulo*, functional readout to identify E3 ligase hotspots that dictate degrader efficacy. We leverage human haploid genetics to describe how the resistance frequency and mutation types are different for PROTACs hijacking the non-essential SR CRBN and the essential SR VHL. Further focusing on the two SRs, we show that cellular reconstitution of loss of function clones with deep mutational scanning (DMS) libraries enables the scalable identification of functional hotspots. Variant enrichment under degrader selection revealed neo-substrate and ternary-complex specific, as well as chemotype selective functional hotspots for CRBN and VHL. Mechanistically, specific hotspots often converge on defects in ternary complex assemblies, as shown by biophysical assays using fully recombinant proteins. Integrating the resulting functional landscapes with crystallographic structural data shows that some of the validated hotspots can be rationalized based on the observed ternary complex structure, implying high complementarity of both approaches. In other cases, existing structures fail to resolve the often profound, functional differences. This indicates that DMS provides a resolution that is partially outside the reach of structural characterization. Finally, integration of DMS data with available clinical data suggests that functional CRBN hotspots are mutated in multiple myeloma patients relapsing from treatment with lenalidomide and pomalidomide, two CRBN-based molecular glue degraders.

In sum, we present a fast, scalable, and experimentally widely accessible methodology that supports the dissection of functional determinants of drug-induced neo-substrate recognition and degradation. This empowers the characterization and optimization of small-molecule degraders and informs on resistance mechanism of putative clinical relevance.

## Results

### Resistance Mechanisms differ between CRBN- and VHL PROTACs

**Extended Data Figure 1:**
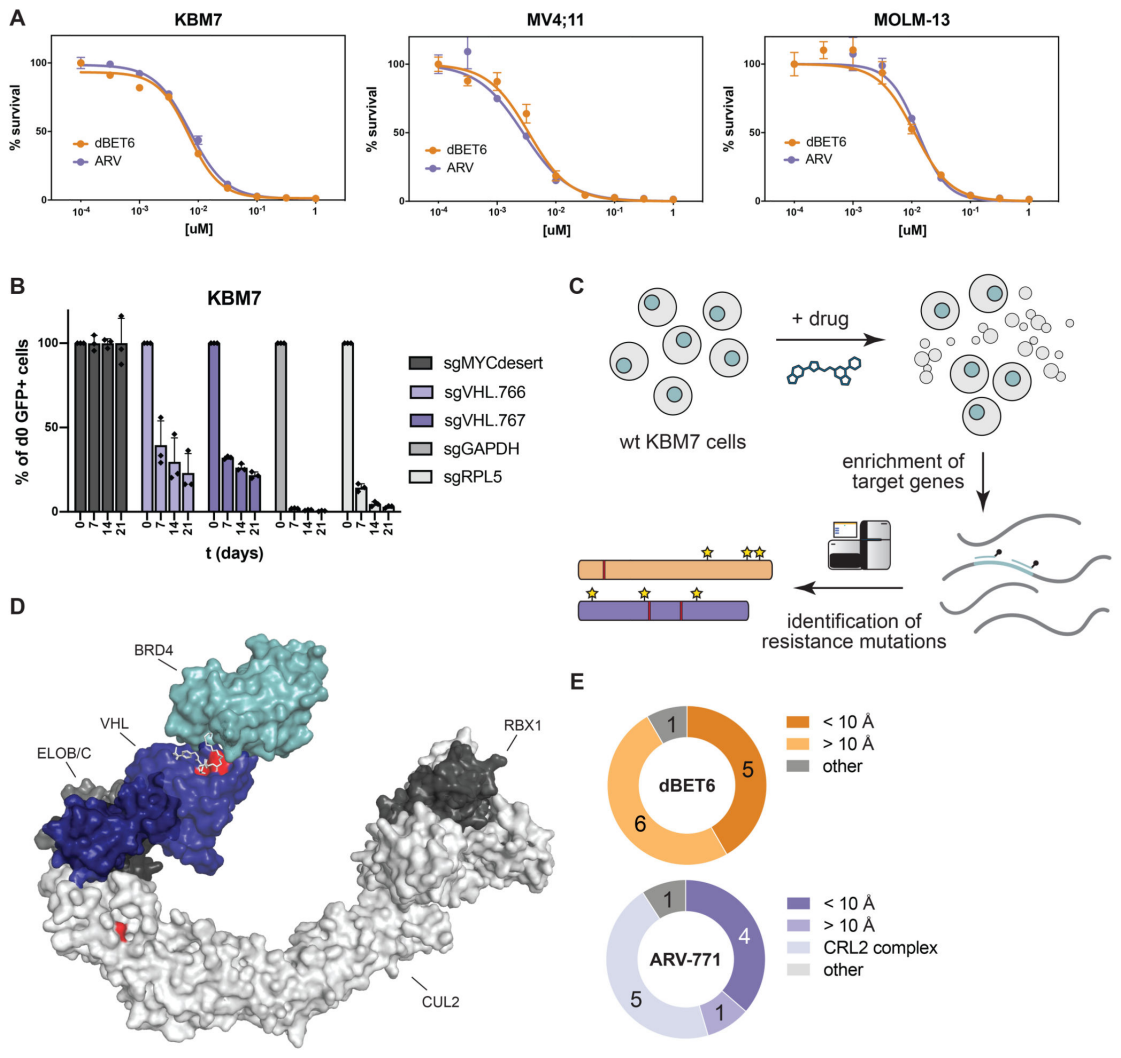
(A) Dose-resolved, normalized viability after 3 d treatment (dBET6 or ARV-771) in KBM7, MV4;11 and MOLM-13 cells. Mean ± s.e.m.; n = 3 independent treatments. (B) Histogram depicting growth competition experiments. WT control KBM7 cells were mixed with mCherry and Cas9 expressing KBM7 cells harboring sgRNAs against the indicated genes. Pools were flow cytometry quantified at days 0, 7, 14 and 21 and mCherry percentages were normalized to day 0 percentage and to a non-targeting control sgRNA (sgMYCdesert). Data represents mean ± s.d. of n = 3 biological replicates. (C) Scheme of targeted hybrid-capture approach coupled to next-generation sequencing to identify mutations in spontaneously resistant cells. (D) Structure depiction of the CUL2-VBC-MZ1-BRD4 complex (PDBs: 5N4W, 5T35). Residues marked in red were identified in hybrid capture analysis. See also Figure 1 and Supplementary Dataset. (E) Number of spontaneous degrader resistance alterations in the substrate receptor (CRBN, VHL, colored) binned by their distance to the degrader binding site. See also Figure 1D and Supplementary Dataset.

**Figure 1:**
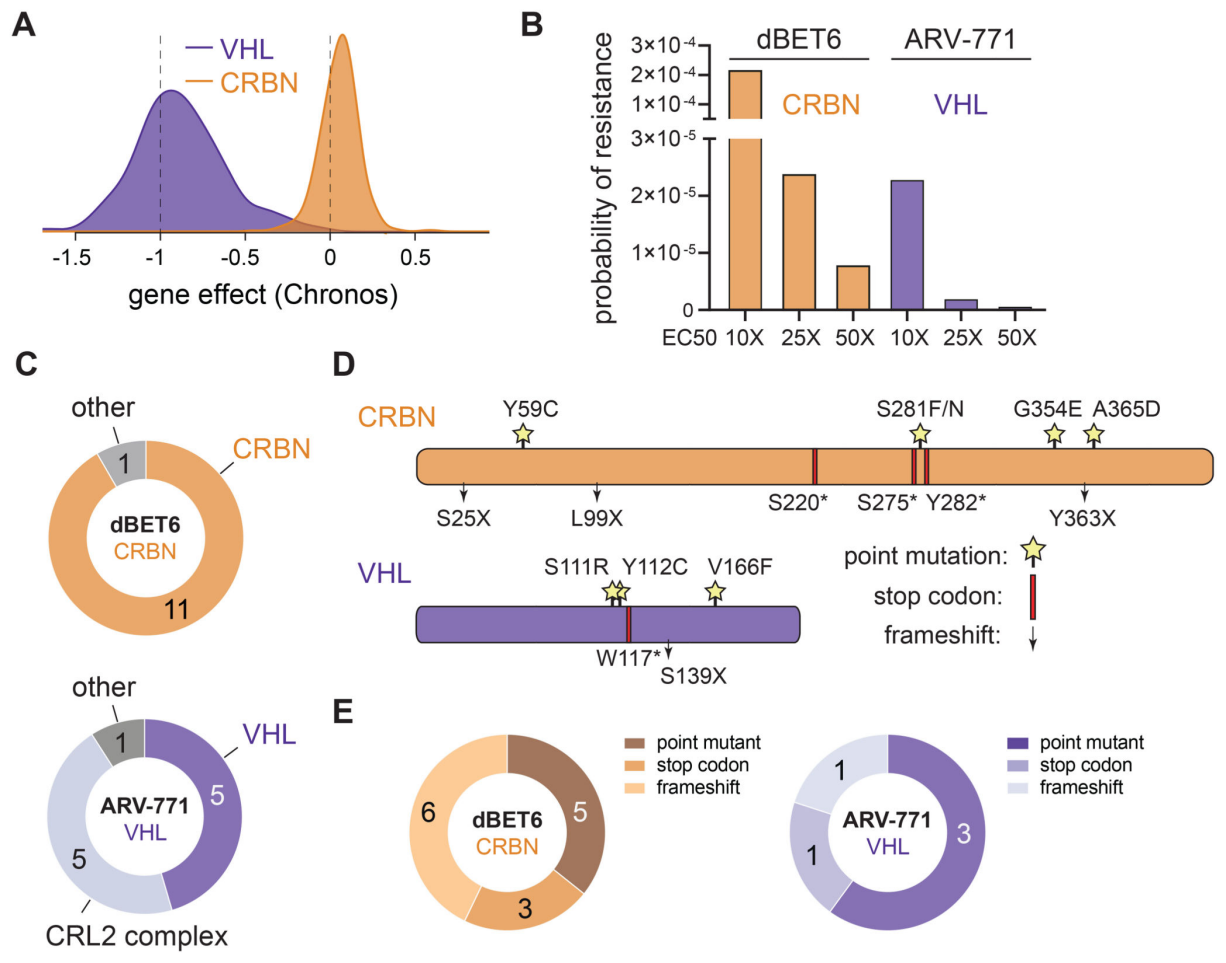
Quantitative and Qualitative Differences in Degrader Resistance (A) Distribution of CRBN and VHL deletion effect (Chronos) across 1070 cancer cell lines. Data taken from Broad Institute DepMap Consortium (22Q1, public). (B) Probability of resistance in KBM7 cells treated at 10, 25 and 50 times EC50 with CRBN (dBET6) and VHL (ARV-771) based BET-bromodomain targeting PROTACs. (C) Number of spontaneous degrader resistance mutations in the substrate receptor (CRBN, VHL), the corresponding Cullin-RING-Ligase (CRL) complex and other degradation associated genes identified in KBM7 cells treated with dBET6 and ARV-771 (10, 25 and 50 times EC50) for 8 to 14 days via targeted hybrid-capture and next-generation sequencing (see also Extended Data Fig. 1). (D) Depiction of CRBN and VHL mutations identified by hybrid-capture sequencing in drug-resistant cell pools. Stars indicate point mutations. Red bars indicate premature stop codons. Arrows indicate frameshift mutations. (E) Number of spontaneous degrader resistance alterations in the substrate receptor (CRBN, VHL) binned according to mutation type (point mutations, gained stop codons, frameshifts). See also Extended Data Fig. 1 and Supplementary Table 1 and Supplementary Dataset.

Conceptually, complete loss-of-function of an essential gene poses a disadvantageous mechanism to evade selective pressure elicited by a drug. Here, we focused our efforts on the two most-commonly adopted SRs CRBN and VHL, both of which are hijacked by degraders in clinical use or entering clinical trials^7^. Mining publicly available data from the DepMap Consortium, *CRBN* presents as a non-essential gene across 1070 cell lines that were profiled via genome-scale CRISPR/Cas9 knockout screens (Fig. 1A)^24^. Despite its well-established role as a tumor suppressor in renal carcinoma^25^, *VHL* proved essential in 935 of the profiled cell lines. To determine if this difference in essentiality is reflected in differential resistance acquisition, we focused on two BET Bromodomain targeting PROTACs: dBET6 (*CRBN*-based) and ARV-771 (*VHL*-based) that have matched cellular potency, including in the near-haploid human leukemia cell line KBM7 (Extended Data Fig. 1A)^26,27^. First, we validated the essentiality of *VHL* in KBM7 cells by CRISPR/Cas9-mediated disruption of *VHL* in competitive growth assays (Extended Data Fig. 1B). Previous studies have shown that *CRBN* loss is inconsequential for KBM7 proliferation^15^. KBM7 cells, which are a frequently used tool to study mechanisms of drug resistance are thus a valid model to capture the overall essentiality profile of both ligases. ^28–30^. We next determined the resistance frequency in KBM7 cells via outgrowth experiments after single dose treatments with either dBET6 or ARV-771. Despite their matched cellular efficacy, occurrence of resistant clones was ten-fold increased after exposure to dBET6 compared to ARV-771 (Fig. 1B). To identify mutations underpinning these quantitative differences, we isolated pools of drug-resistant clones and subjected them to a hybrid capture based targeted sequencing approach (Extended Data Fig. 1C). This strategy covers all members of the respective CRL ligase complexes, CRL regulatory proteins as well as the recruited POIs (Supplementary Table 1). In dBET6 resistant cells, we identified the majority of disruptive alterations directly in *CRBN*, while other members of the CRL4^CRBN^ ligase complex were not affected (Fig. 1C, Supplementary Dataset). In contrast, cells resistant to ARV-771 featured a lower proportion of genetic defects directly in *VHL* and an equal number of alterations in various other components of the CRL2^VHL^ complex, such as *CUL2* and *ELOB*. We found a higher fraction (55 %) of frameshifts and gained stop-codons in *CRBN*. In contrast, the majority (60%) of alterations in *VHL* were missense point mutations (Fig. 1D and E, Supplementary Dataset). Together, these data implicate the SR as the most frequently mutated CRL component in degrader-resistant clones. However, both the frequency and the type of alterations appear to be influenced by the essentiality of the co-opted SR. In case of hijacking VHL, the fitness costs associated with directly mutating the essential SR favors mutations acquired in other complex members, such as *CUL2*. Supporting these results, loss of *CUL2* has previously been reported as an acquired resistance mechanism to VHL-based PROTACs in OVCAR8 cells^16^.

### DMS Identifies Functional Hotspots of General Relevance

**Figure 2:**
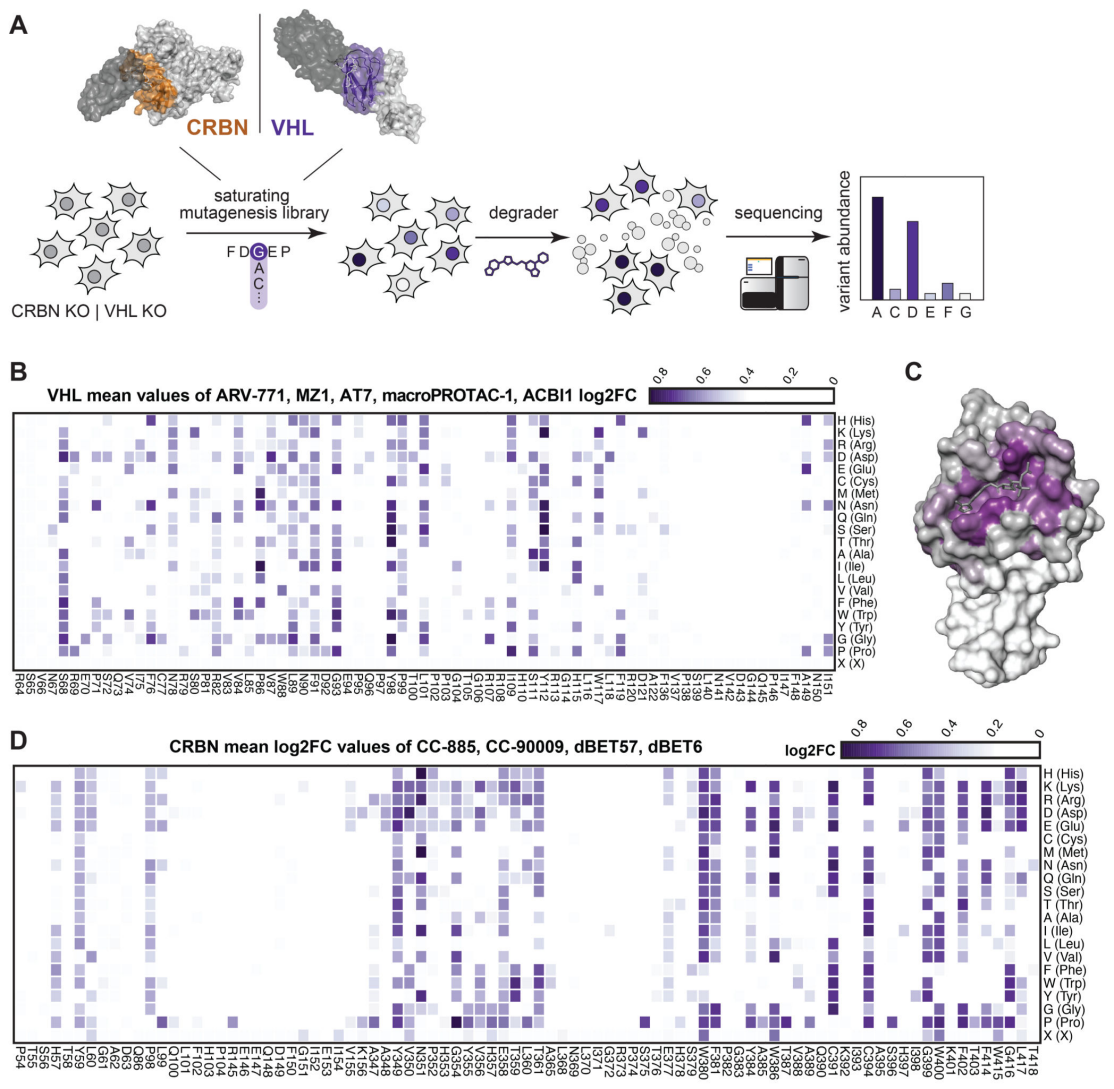
Deep Mutational Scanning Locates Functional Hotspots of General Relevance in the Degrader Binding Pocket (A) Deep-mutational-scanning approach to identify resistance conferring CRBN and VHL mutants in 10 Å proximity (colored ochre and purple) of the ligand binding site via next-generation sequencing. (B) Heatmap depicting mean log2 fold-enrichment of VHL mutations normalized to maximum log2 fold-changes vs. DMSO across 5 degraders (500 nM ARV-771, 500 nM MZ1, 500 nM AT7, 2 μM macroPROTAC-1, 2 μM ACBI1) treated for 7 days. n = 2 independent measurements. (C) Surface structure of VHL bound by VHL Ligand VH032, PDB 4W9H^50^. Median log2 fold-enrichment of all VHL mutations over DMSO across 5 degrader treatments (see Fig. 2B) is mapped in purple to dark grey onto positions mutated in the library. (D) Heatmap depicting mean log2 fold-enrichment of CRBN mutations normalized to maximum log2 fold-changes vs. DMSO across 4 degraders (500 nM dBET6, 500 nM dBET57, 500 nM CC-90009, 500 nM CC-885) treated for 7 days. n = 3 independent measurements. See also Extended Data Fig. 2 and 3.

Many point mutations were identified proximal to the degrader binding pocket and the predicted neo-substrate interface, highlighting the importance of the SR in orchestrating ternary complex formation (Extended Data Fig. 1D and E). To systematically investigate the surface topology of both SRs at an amino acid resolution, we designed DMS libraries for all VHL and CRBN positions in proximity of the degrader binding site (< 10 Å, Fig. 2A) covering 1442 and 1738 different variants, respectively. Noteworthy, DMS strategies have previously been successfully employed to investigate functional relationships between small molecules and target proteins^31,32^. Here, we surmised that when coupled with a selectable readout, variant libraries could inform on functional hotspots in the respective SR. Considering the specific molecular architecture of the drug-induced ternary complex, such hotspots could either be conserved over different degraders, or specific for a particular compound.

**Extended Data Figure 2:**
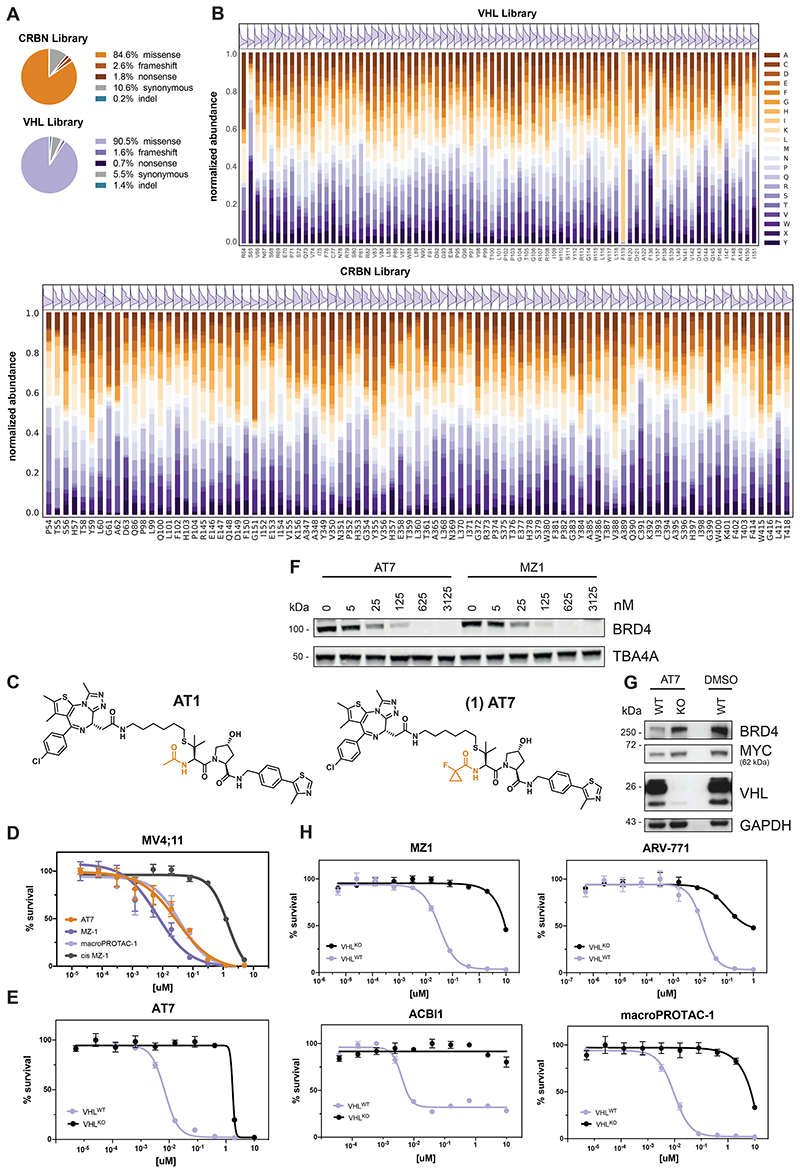
(A) Pie charts depicting the distribution of different alterations identified by sequencing the mutational scanning libraries for CRBN (top) and VHL (bottom). (B) Stacked bar graphs and density distributions of residue wise normalized abundance of mutants identified in the DMS libraries for VHL (top) and CRBN (bottom). (C) Chemical structure comparison of the degraders AT1 and AT7. (D) Dose-resolved, normalized viability after 3 d treatment with MZ-1, macroPROTAC-1, cis MZ-1 (a non VHL binding control of MZ-1 or AT7 in MV4;11 cells. Mean ± s.e.m.; n = 3 independent treatments. (E) Dose-resolved, normalized viability after 3 d treatment (AT7) in RKO VHL^-/-^ cells with over-expression of VHL^WT^. Mean ± s.e.m.; n = 3 independent treatments. (F) Protein levels in HeLa cells treated with MZ-1 or AT7 (18h, indicated concentration). Representative images of n = 2 independent measurements. (G) Protein levels in RKO VHL^-/-^ cells with over-expression of VHL^WT^ treated with DMSO or AT7 (60 nM, 2 h). Representative images of n = 2 independent measurements. (H) Dose-resolved, normalized viability after 4 d treatment (ACBI1) and 3 d treatment (ARV-771, MZ-1, macroPROTAC-1) in RKO VHL^-/-^ cells with over-expression of VHL^WT^. Mean ± s.e.m.; n = 3 independent treatments.

**Extended Data Figure 3:**
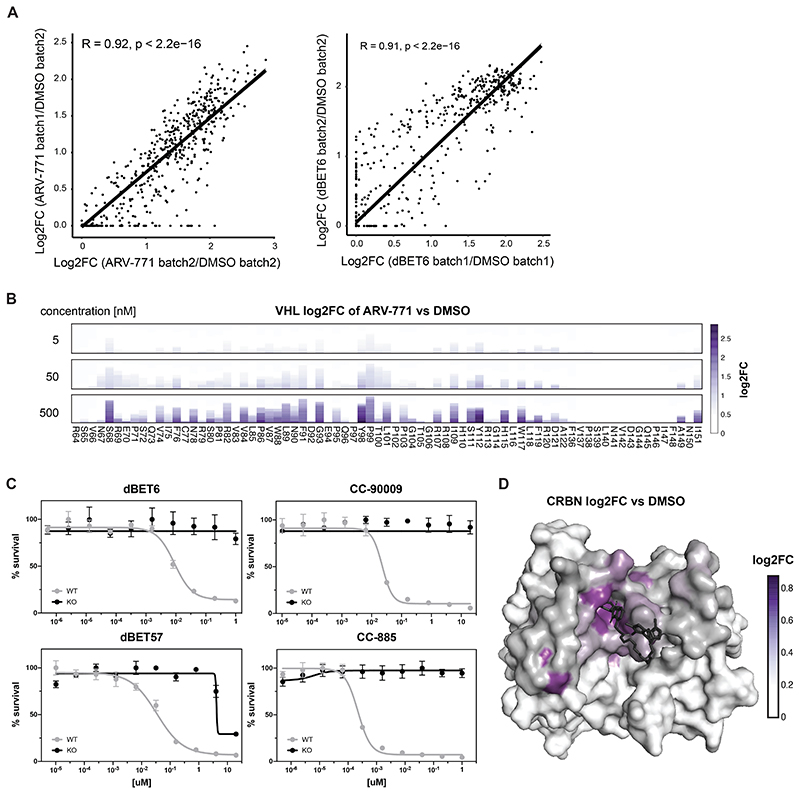
(A) Scatter plot depicting log2 fold-enrichment between different batch mutational scanning resistance measurements of VHL (500 nM ARV-771) or CRBN mutations (500 nM dBET6) normalized to DMSO after 7-day treatment. The rank-based measure of association was estimated via Spearman’s rho statistic and reported P-values were calculated via asymptotic two-sided *t* approximation without adjustments for multiple comparisons. (B) Stacked bar graphs of log2 fold-enrichment of VHL mutants normalized to DMSO treated with the indicated concentrations of ARV-771 for 7 days. n = 2 independent measurements. (C) Dose-resolved, normalized viability after 3 d treatment with dBET6, CC-90009, dBET57 or CC-885 in RKO CRBN^-/-^ cells with over-expression of CRBN^WT^. Mean ± s.e.m.; n = 3 independent treatments. (D) Surface structure of CRBN bound by dBET6 (PDB 6BOY). Median log2 fold-enrichment of all CRBN mutations over DMSO across 4 degrader treatments (see Figure 2D) is mapped in purple to dark grey onto positions mutated in the CRBN library.

To initially ensure quality control, we sequenced the prepared libraries and mostly identified expected missense variants (Extended Data Fig. 2A). Furthermore, an even distribution of possible substitutions was present for almost all residues (Extended Data Fig. 2B, see also Methods section). Next, to establish proof of concept, we reconstituted *VHL*-deficient RKO colon carcinoma cells (*VHL*^-/-^), with the corresponding variant library. Selective pressure was applied through treatment with five different VHL-based PROTACs for seven days. The assayed PROTACs either target BRD4 and related BET bromodomain family proteins (MZ1^33^, ARV-771^26^ and macroPROTAC-1^34^), or the BAF complex subunits SMARCA2/4 for degradation (ACBI1^35^). To sample greater diversity of PROTAC exit vectors and linkers, we additionally designed AT7 (**1**) as an analogue of the previously disclosed AT1^10^. While AT7, similar to AT1, branches out of the VHL ligand *tert*-butyl group via a thioether linker, it bears a fluoro-cyclopropyl capping group instead of the methyl group of AT1 (Extended Data Fig. 2C). This capping group is known to enhance the binding affinity to VHL as well as aid new PPIs within PROTAC ternary complexes^35,36^. In cellular assays, AT7 exhibited potent cytotoxicity and BRD4 degradation (Extended Data Fig. 2D to G). All degraders blocked the proliferation of RKO cells in a *VHL* dependent manner, enabling sufficient selective pressure (Extended Data Fig. 2E and H). After the selection, VHL variants that conferred a proliferative advantage were identified via next generation sequencing by their enrichment over an unselected (vehicle-treated) population. We initially validated the robustness of this experimental setup between biological replicates (*R* = 0.92, Extended Data Fig. 3A). Averaging log_2_ fold-enrichment for each mutation across all 5 degraders generated a map of consensus VHL hotspots (Fig. 2B). As expected, residues of shared relevance primarily localized to the binding pocket of the closely related VHL ligands of the various assayed PROTACs (Fig. 2C). Hotspots were highly robust and conserved over a wide concentration range (Extended Data Fig. 3B).

We next aimed to expand our analyses to CRBN, assaying two BET PROTACs (dBET6, dBET57), and two molecular glue degraders (CC-885, CC-90009) degrading GSPT1 (Fig. 2D and Extended Data Fig. 3C)^37,38^. As observed for VHL, functional CRBN hotspots that were enriched across all tested degraders localized to the glutarimide (ligand-) binding pocket. (Extended Data Fig. 3D). In sum, the presented deep mutational scanning approach empowered the robust and reproducible identification of functional hotspots of general relevance over different degrader modalities, ligases and neo-substrates.

### Characterizing Neo-Substrate Specific Functional VHL Hotspots

**Extended Data Figure 4:**
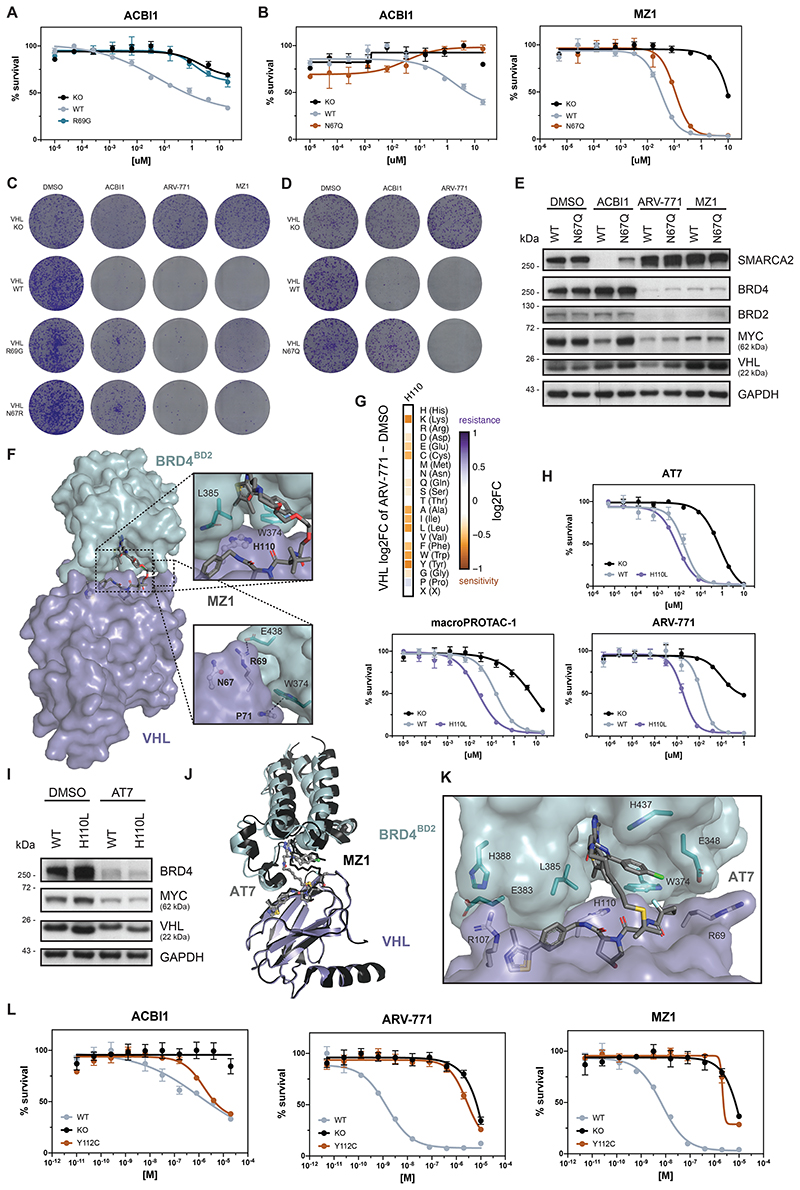
(A and B) Dose-resolved, normalized viability after 4 d treatment (ACBI1) and 3 d treatment (MZ-1) in RKO VHL^-/-^ cells with over-expression of VHL^WT^, VHL^R69G^ or VHL^N67Q^. Mean ± s.e.m.; n = 3 independent treatments. (C and D) Depiction of clonogenic assays via crystal violet staining. RKO VHL^-/-^ cells with over-expression of VHL^WT^, VHL^R69G^, VHL^N67R^ or VHL^N67Q^ were treated for 10 days at EC90 of the degrader (2.5 uM ACBI1, 50 nM ARV-771, 75 nM MZ-1). (E) Protein levels in RKO VHL^-/-^ cells with over-expression of VHL^WT^ or VHL^N67Q^ treated with DMSO, MZ-1 (75 nM, 2 h), ARV-771 (50 nM, 2 h) or ACBI1 (2.5 uM, 4 h). Representative images of n = 2 independent measurements. (F) Cocrystal structure of MZ-1 in a ternary complex with VHL-ElonginC-ElonginB and BRD4^BD2^ (PDB: 5T35). (G) Heatmap depicting differential log2 fold-enrichment of the VHL^H110^ mutations normalized to DMSO after treatment with ARV-771 (500 nM, 7d). n = 2 independent measurements. (H) Dose-resolved, normalized viability after 3d treatment AT7 (top), macroPROTAC-1 (bottom, left) or ARV-771 (bottom, right) in RKO VHL^-/-^ cells with over-expression of VHL^WT^ or VHL^H110L^. Mean ± s.e.m.; n = 3 independent treatments. (I) Protein levels in RKO VHL^-/-^ cells with over-expression of VHL^WT^ or VHL^H110L^ treated with DMSO or AT7 (60 nM, 2 h). Representative images of n = 2 independent measurements.

**Figure 3:**
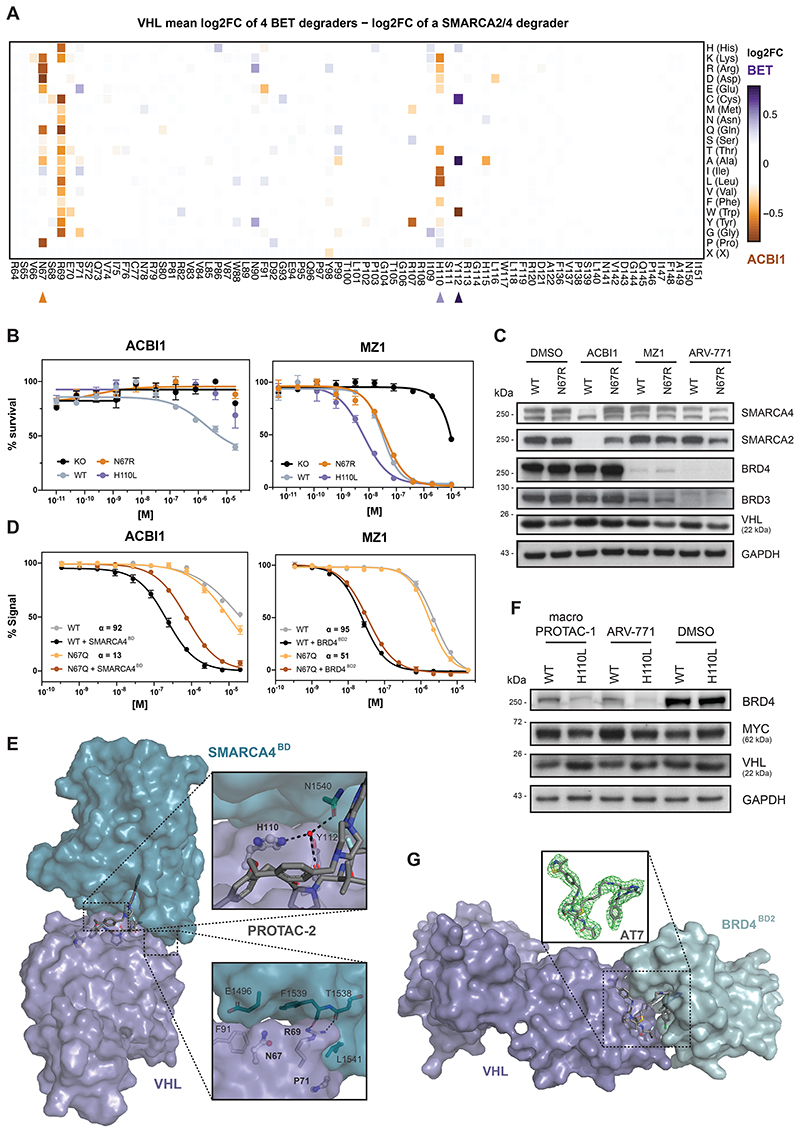
Functional VHL Hotspots Identified by DMS Show Neo-Substrate Dependent Resistance and Sensitivity to PROTAC Treatment (A) Heatmap depicting differential log2 fold-enrichment of VHL mutations normalized to maximum log2 fold-changes vs. DMSO between the mean of 4 BET PROTACs (500 nM ARV-771, 500 nM MZ1, 500 nM AT7, 2 μM macroPROTAC-1) and the SMARCA2/4 PROTAC ACBI1 (2 μM). Treated for 7 days; n = 2 independent measurements. (B) Dose-resolved, normalized viability after 4 d treatment (ACBI1, left) and 3 d treatment (MZ1, right) in RKO VHL^-/-^ cells with over-expression of VHL^WT^, VHL^N67R^ or VHL^H110L^. Mean ± s.e.m.; n = 3 independent treatments. (C) Protein levels in RKO VHL^-/-^ cells with over-expression of VHL^WT^ or VHL^N67R^ treated with DMSO, ACBI1 (2.5 μM, 4h), MZ1 (75 nM, 2h) and ARV-771 (50 nM, 2h). Representative images of n = 2 independent measurements. (D) Fitted curves from fluorescence polarization competition assays measuring displacement of a VHL peptide from either WT or mutant VCB protein by ACBI1 (left) or MZ1 (right) in the presence or absence of saturating concentrations of SMARCA4^BD^ or BRD4^BD2^ protein. Mean ± s.d.; n = 3 technical replicates. (E) Cocrystal structure of PROTAC-2 (close analogue to ACBI1) in a ternary complex with VHL-ElonginC-ElonginB and SMARCA4^BD^ (PDB 6HAX). (F) Protein levels in RKO VHL^-/-^ cells with over-expression of VHL^WT^ or VHL^H110L^ treated with DMSO, macroPROTAC-1 (250 nM, 2h), ARV-771 (12.5 nM, 90 min). Representative images of n = 2 independent measurements. (G) Cocrystal structure of AT7 in a ternary complex with VHL-ElonginC-ElonginB and BRD4^BD2^ solved to a resolution of 3.0 Å. The omit difference electron density map (Fo−Fc) is shown in green in the inset panel, superimposed around AT7 and contoured at 3σ. See also Extended Data Fig. 4.

To focus the resolution towards unique, potentially substrate-specific, hotspots, we compared enrichments for the SMARCA2/4 PROTAC ACBI1^35^ to the average enrichment of all assayed BET degraders (Fig. 3A). This allowed identification of the functional hotspots VHL^N67^, VHL^R69^ and VHL^H110^, which appear to be specifically required to sustain the activity of ACBI1, while they seem inconsequential for the tested BET PROTACs. In support of this, published co-crystal structures and TR-FRET data previously validated the importance of VHL^R69^ in SMARCA2^BD^ recognition within the ternary complex^35^. To further confirm the specificity of these hotspots, we generated single point mutant reconstitutions in VHL^-/-^ RKOs and assessed cellular fitness following drug treatments (Fig. 3B and Extended Data Fig. 4A to D). Indeed, mutating VHL^N67^ rescued the efficacy of ACBI1 without modulating the efficacy of BET PROTACs. These differences functionally converge on an altered neo-substrate degradation. In cells expressing a VHL^N67^ mutant, ACBI1 failed to induce SMARCA2/4 degradation at conditions where profound degradation is observed in isogenic VHL^WT^ cells. In contrast, BRD3/4 destabilization by the assayed BET degraders was unaffected by VHL^N67^ mutation (Fig. 3C and Extended Data Fig. 4E). Given the positioning of VHL^N67^ at the VHL:SMARCA2/4 binding interface yet not in direct contact with the PROTAC itself (Fig. 3E), we surmised that the lack of SMARCA2/4 degradation with the VHL^N67^ mutant might mechanistically be caused by defects in integrity and stability of the ternary complex. To address this, we established fluorescence polarization experiments assessing the extent to which ternary complex formation and cooperativity of the induced tripartite binding is affected by the VHL mutation. Specifically, PROTAC binding to purified *wildtype*, or mutated VHL-ElonginC-ElonginB (VCB) was measured in absence and presence of recombinant SMARCA4^BD^ or BRD4^BD2^. This led us to identify that mutations in VHL^N67^ (here VHL^N67Q^) decrease the ternary complex affinity and cooperativity of ACBI1 binding to SMARCA4^BD^ by ~7-fold (Fig. 3D). In contrast, the affinity and cooperativity of the VHL:MZ1 binary complex to BRD4^BD2^ was largely unaffected by mutations in VHL^N67^ (within 2-fold those of wild-type, Fig. 3D). In the ternary crystal structure of a close ACBI1 analogue in complex with VCB and SMARCA4^BD^ (PDB: 6HR2), the side chain of VHL^N67^ sits against the protein-protein interface sandwiched between VHL^R69^ and VHL^F91^ (Fig. 3E). While the asparagine side chain does not interact directly with SMARCA4, neighboring residues contribute PPIs. Therefore, any unfavorable VHL^N67^ changes can negatively impact ternary complex formation. In contrast, in the ternary crystal structures of BET degraders such as MZ1^10^ (PDB: 5T35), VHL^N67^ is distal from the induced PPI and does not impact ternary complex formation, explaining why VHL^N67^ was not a hotspot for the assayed BET degraders (Extended Data Fig. 4F).

Of note, the dose range and experimental setup of our DMS strategy was geared to reveal resistance-causing mutations. Accordingly, DMS also identified VHL^H110L^ as a mutation that causes resistance to ACBI1, which we could validate via single point mutant reconstitutions (Fig. 3A and B). Intriguingly, this mutation simultaneously sensitized cells to treatment with certain BET PROTACs, such as MZ1 (5-fold) or ARV-771 (6-fold, Fig. 3B and Extended Data Fig. 4G and H). This highlights VHL^H110L^ as potentially “versatile” in nature, meaning that its effect can be either sensitizing, neutral or resistance-causing, based on the assayed drug. Intriguingly, this sensitization effect was not uniform for all tested BET PROTACs. ARV-771, MZ1 and the macrocyclic BET degrader macroPROTAC-1^34^ showed higher levels of augmentation, while sensitization for AT7 appeared attenuated (Extended Data Fig. 4H). This was further supported by BRD4 degradation upon PROTAC treatment in VHL^H110L^ expressing cells (Fig. 3F and Extended Data Fig. 4I). In an effort to understand these nuanced functional effects, we solved the cocrystal structure of the ternary complex between BRD4^BD2^: AT7:VCB to a resolution of 3.0 Å (Fig. 3G). Remarkably, despite the unique linker geometry and increased lipophilicity, the ternary structure of AT7 proved largely conserved in relation to the cocrystal ternary structures of both MZ1^10^ and macroPROTAC-1^34^. While there are no discernable changes in key PPIs, the entire bromodomain shifts laterally (r.m.s.d. of 2.1 Å) to accommodate the new PROTAC molecular architecture (Extended Data Fig. 4J). As in the structure of MZ1 and macroPROTAC-1, VHL^H110^ sits underneath the bromodomain in a hydrophobic patch formed by BRD4^W374^, BRD4^L385^ and the di-methyl thiophene of the JQ1 warhead (Extended Data Fig. 4F and K). It is therefore structurally plausible that a mutation of VHL^H110^ to a hydrophobic residue such as leucine at this position could have a beneficial impact on ternary binding affinity by enhancing favorable hydrophobic interactions. In contrast to the role VHL^H110^ plays in the BET ternary structures, the SMARCA4 ternary structure reveals an alternative side-chain conformation. Here VHL^H110^ points back towards the VHL ligand and forms a bridging hydrogen bond to a highly coordinated water trapped at the core of the ternary structure (Fig. 3E). Mutation of this histidine to a lipophilic residue, such as leucine, would drastically change this water environment. Additionally, the substitution of the planar side chain of histidine for the bulky branched side chain in leucine is likely to cause a steric clash at closely located PPIs.

Finally, our DMS analysis highlighted the functional hotspot VHL^Y112^, which was also found mutated in our assessment of spontaneous resistance mechanisms (Fig. 1D and 3A). Intriguingly, the mutant VHL^Y112C^ elicited selective resistance to BET degraders while having nearly no effect on ACBI1 potency (Extended Data Fig. 4L). Together, this showcases how our comparative analysis of systematic amino acid mutation can elucidate functional hotspots that modulate drug-induced degradation in a neo-substrate selective manner. Many of the functional consequences of individual mutations can be rationalized from a structural perspective. However, as exemplified via VHL^H110L^, DMS data can provide a layer of functional resolution that is not immediately obvious from structure-centric approaches.

### VHL Resistance Hotspots Are Specific to Distinct Degraders

**Extended Data Figure 5:**
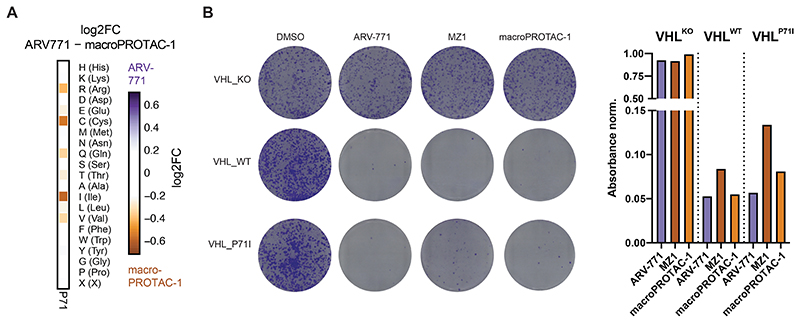
(A) Heatmap depicting differential log2 fold-enrichment of the VHL^P71^ mutations normalized to DMSO between treatment with ARV-771 (500 nM, 7d) and macroPROTAC-1 (2 uM, 7d). n = 2 independent measurements. (B) Depiction (left) and quantification (right) of clonogenic assays via crystal violet staining. RKO VHL^-/-^ cells with over-expression of VHL^WT^ or VHL^P71I^ were treated for 10 days at EC90 of the degrader (50 nM ARV-771, 75 nM MZ-1, 1 uM macroPROTAC-1).

**Figure 4:**
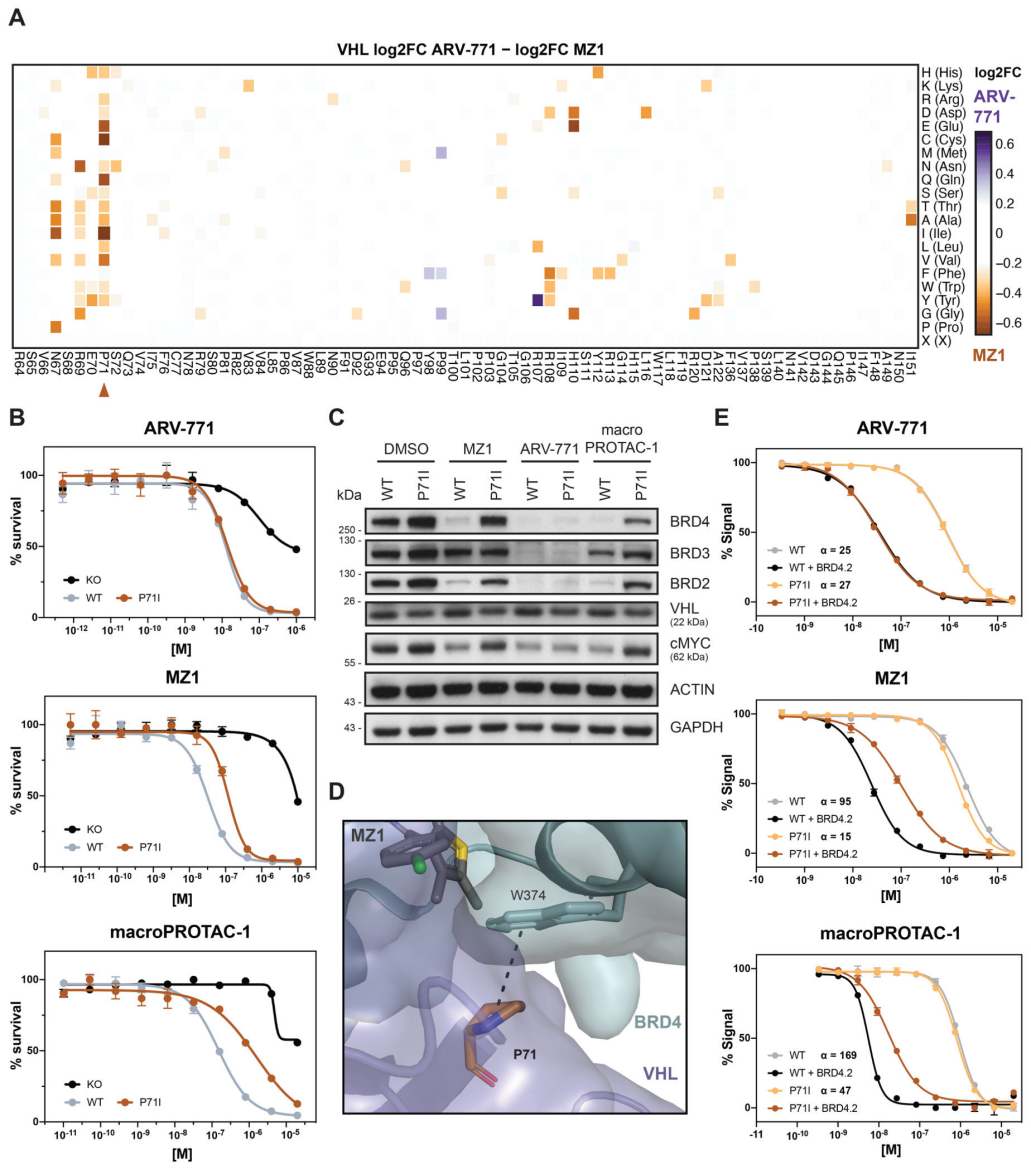
VHL^P71^ is a Functional Hotspot for Degrader Specific Resistance (A) Heatmap depicting differential log2 fold-enrichment of VHL mutations normalized to maximum log2 fold-changes vs. DMSO between BET bromodomain targeting PROTACs ARV-771 (500 nM, 7d) and MZ1 (500 nM, 7d). n = 2 independent measurements. (B) Dose-resolved, normalized viability after 3d treatment with ARV-771 (top), MZ1 (center) and macroPROTAC-1 (bottom) in RKO VHL^-/-^ cells with over-expression of VHL^WT^ or VHL^P71I^. Mean ± s.e.m.; n = 3 independent treatments. (C) Protein levels in RKO VHL^-/-^ cells with over-expression of VHL^WT^ or VHL^P71I^ treated with DMSO, MZ1 (37.5 nM, 90 min), ARV-771 (25 nM, 90 min) or macroPROTAC-1 (480 nM, 90 min). Representative images of n = 2 independent measurements. (D) Cocrystal structure of MZ1 in a ternary complex with VHL-ElonginC-ElonginB and BRD4^BD2^ (PDB 5T35) depicting an interaction between VHL^P71^ and the BRD4^WPF^ shelf. (E) Fitted curves from fluorescence polarization competition assays measuring displacement of a VHL peptide from either WT or mutant VCB protein by PROTACs in the presence or absence of saturating concentrations of partner protein. Mean ± s.d.; n = 3 technical replicates See also Extended Data Fig. 5.

We next set out to identify differential hotspots among degraders with an overlapping neo-substrate spectrum, as exemplified by the tested BET PROTACs. Comparative analysis of DMS enrichments revealed that VHL^P71^ is selectively critical for the efficacy of MZ1 and macroPROTAC-1(Fig. 4A and Extended Data Fig. 5A). These findings were subsequently validated in individual reconstitution experiments (Fig. 4B, C and Extended Data Fig. 5B). Previous structural elucidation of the MZ1-induced ternary complex has revealed a role of VHL^P71^ by extending the BRD4^WPF^ shelf through additional CH-pi interactions with BRD4^W374^ (Fig. 4D)^10^. This interfacial positioning of P71 prompted us to again investigate whether the underlying molecular mechanism is connected to altered assembly affinity of the ternary complex. Fluorescence polarization assays indicated that the binding cooperativity between MZ1, BRD4^BD2^ and VCB is significantly (6-7 fold) affected upon introducing the VHL^P71I^ mutation (Fig. 4E). A similar effect was also observed for macroPROTAC-1. In contrast, the cooperativity of ARV-771-induced ternary complex formation is not affected (Fig. 4E), suggesting that the ARV-771-induced ternary complex features a unique architecture that is likely distinct from the architecture observed for MZ1.

In sum, we show that DMS empowers a functional segregation of different drug-induced, ternary complexes that involve identical neo-substrates. This is best exemplified by complexes induced by the BET protein degrader ARV-771, which has, intriguingly, at least in our hands so far proven intractable to structural exploration via crystallography.

### Functional CRBN Hotspots Are Mutated in Relapsing Patients

Next, we turned our focus to CRBN, the only E3 ligase that to date is clinically validated via the FDA-approved molecular glue degrader lenalidomide and related analogs (collectively often referred to as immunomodulatory drugs, IMiDs). This gives us the chance to identify functional hotspots that differentiate between the two paradigmatic small-molecule degrader modalities: heterobifunctional PROTACs and monovalent molecular glues. Moreover, we hypothesized that DMS might elucidate functional hotspots involved in resistance mechanisms that are of clinical relevance.

**Extended Data Figure 6:**
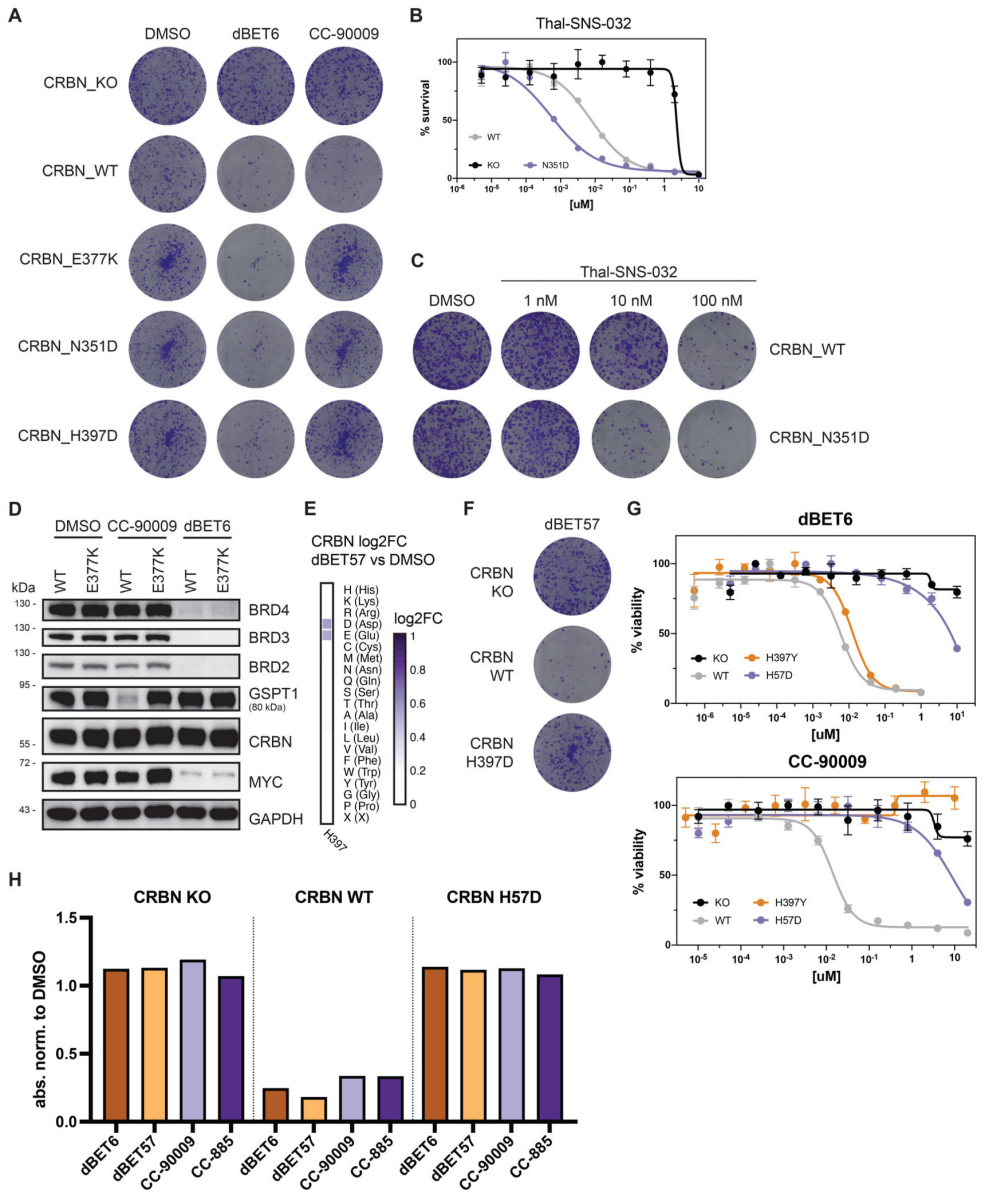
(A, C and F) Depiction of clonogenic assays via crystal violet staining. RKO CRBN^-/-^ cells with over-expression of CRBN^WT^, CRBN^E377K^, CRBN^N351D^ or CRBN^H397D^ were treated for 10 days with DMSO, 30 nM dBET6, 60 nM CC-90009, 480 nM dBET57 or the indicated concentration of THAL-SNS-032. (B and G) Dose-resolved, normalized viability after 3 d treatment with THAL-SNS-032, dBET6 or CC-90009 in RKO CRBN^-/-^ cells with over-expression of CRBN^WT^, CRBN^N351D^, CRBN^H397Y^ or CRBN^H57D^. Mean ± s.e.m.; n = 3 independent treatments. (D) Protein levels in RKO CRBN^-/-^ cells with over-expression of CRBN^WT^ or CRBN^E377K^ treated with DMSO, CC-90009 (50 nM, 6 h) or dBET6 (15 nM, 2 h). Representative images of n = 2 independent measurements. (E) Heatmap depicting differential log2 fold-enrichment of CRBN^H397^ mutations normalized to DMSO with dBET57 treatment (500 nM, 7d). n = 3 independent measurements. (H) Quantification of clonogenic assays via crystal violet extraction and measurement of absorption at 590 nM. RKO CRBN^-/-^ cells with over-expression of CRBN^WT^ or CRBN^H57D^ were treated for 10 days with DMSO, 30 nM dBET6, 60 nM CC-90009, 480 nM dBET57 or 0.6 nM CC-885. See also Figure 5.

**Figure 5:**
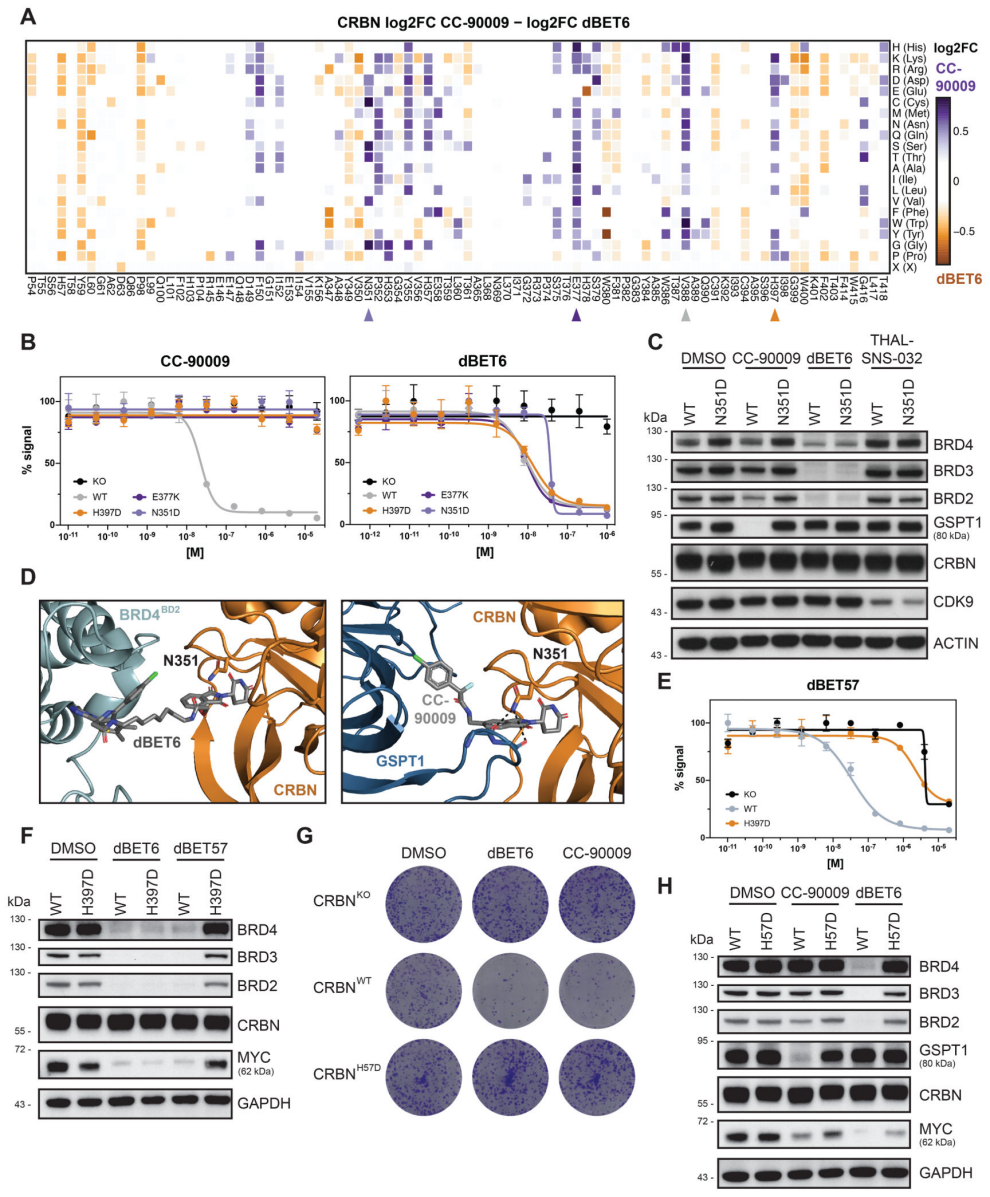
Functional CRBN Hotspots Show Degrader Selectivity and are Mutated in Refractory Multiple Myeloma Patients (A) Heatmap depicting differential log2 fold-enrichment of CRBN mutations normalized to maximum log2 fold-changes vs. DMSO between BET bromodomain targeting PROTAC dBET6 (500 nM, 7 d treatment) and the GSPT1 targeting molecular glue CC-90009 (500 nM, 7 d treatment). n = 3 independent measurements. (B) Dose-resolved, normalized viability after 3 d treatment with CC-90009 and dBET6 in RKO CRBN^-/-^ cells with over-expression of CRBN^WT^, CRBN^E377K^, CRBN^N351D^ and CRBN^H397D^. Mean ± s.e.m.; n = 3 independent treatments. (C, F and H) Protein levels in RKO CRBN^-/-^ cells with over-expression of CRBN^WT^, CRBN^N351D^, CRBN^H397D^ or CRBN^H57D^ treated with DMSO, CC-90009 (50 nM, 6 h), dBET6 (15 nM, 2 h), dBET57 (240 nM, 2 h) or THAL-SNS-032 (200 nM, 2 h). Representative images of n = 2 independent measurements. (D) Cocrystal structure of dBET6 (left) and CC-90009 (right) in a ternary complex with CRBN and BRD4^BD2^ (PDB 6BOY) or GSPT1 (PDB 6XK9) depicting PPIs of CRBN^N351^ and the GSPT1. (E) Dose-resolved, normalized viability after 3 d treatment with dBET57 in RKO CRBN^-/-^ cells with over-expression of CRBN^WT^ and CRBN^H397D^. Mean ± s.e.m.; n = 3 independent treatments. (G) Depiction of clonogenic assays via crystal violet staining. Cells were treated for 10 days at EC90 of the degrader (30 nM dBET6, 60 nM CC-90009). Representative of n = 2 independent measurements. See also Extended Data Fig. 6.

First, we aimed to identify functional CRBN hotspots that show selectivity for molecular glue degraders or PROTACs. We utilized our DMS approach to systematically elucidate functional consequences of CRBN mutations on the efficacy of CC-90009, a clinical-stage molecular glue degrader targeting GSPT1^38^. Comparing CRBN variant enrichment after selection with CC-90009 or the BET PROTAC dBET6^27^ yielded functional CRBN hotspots relevant to either of both classes of degrader modality (Fig. 5A). Among the enriched, glue-selective hotspots, we identified V388 as a key determinant of cellular efficacy of CC-90009. Intriguingly, this site corresponds to position 391 in mouse *Crbn*, which features the critical isoleucine variant that is responsible for the lack of IMiD activity in mouse cells, hence masking the teratogenicity of thalidomide^39^. Of note, DMS analysis resolves the importance of isoleucine, but also indicates that most other substitutions at this position are disruptive. Next, we aimed to expand our survey of functional CRBN hotspots, validating two CC-90009 selective mutants (CRBN^E377K^ and CRBN^N351D^, Fig. 5B and Extended Data Fig. 6A). Interestingly, mutations in CRBN^N351^ showed a highly specific, versatile behavior for different degraders. While cellular expression of CRBN^N351D^ prompted resistance to CC-90009, it was inconsequential for dBET6 (Fig. 5A and B). Simultaneously, it led to a marked sensitization (15-fold shift in EC_50_) to the CDK9-targeting PROTAC THAL-SNS-032^40^ (Extended Data Fig. 6B and C). This differential potency correlated with target degradation levels, highlighting the intricate functional differences that can be uncovered by our DMS analysis (Fig. 5C Extended Data Fig. 6D for CRBN^E377K^). Upon inspection of the ternary structure of CC-90009 (PDB: 6XK9), CRBN^N351^ is found proximal to the protein-protein interface and is in a position to directly interact with the backbone carbonyls of GSPT1 (Fig. 5D). In contrast the structure of dBET6 (PDB:6BOY) reveals that CRBN^351^ is far from the PPI and is thus unlikely to have an effect on ternary complex formation.

We next focused on the CRBN^H397^ position. Interestingly, our DMS data suggested that mutation to only the negatively charged amino acids aspartate or glutamate abrogated the cellular and degradation efficacy of the BET PROTAC dBET57 (Extended Data Fig. 6E). We validated that this mutational effect is not observed for the closely related dBET6 (Fig. 5B, E and F and Extended Data Fig. 6F). Intriguingly, mutations in this position also prompted resistance to molecular glue degraders (Fig. 5A and B and Extended Data Fig. 6G). Furthermore, a mutation in CRBN^H397^ was also identified in a multiple myeloma (MM) patient who presented refractory to IMiD treatment ^41^. Upon closer inspection, several mutations in relapsed patients, such as CRBN^P352S^, CRBN^F381S^ and CRBN^H57D^ overlapped with CRBN hotspots identified by DMS (Fig. 2D, 5G and H and Extended Data Fig. 6G and H)^42^.

Taken together, we report CRBN hotspots that modulate degrader efficacy selectively as well as universally, and which, upon mutation, can either cause resistance or sensitization. Some but not all of these effects could be rationalized via structural investigation. Importantly, DMS also highlighted functional hotspots that are disrupted by mutations in patients relapsing from IMiD treatment.

## Discussion

An essential step in targeted protein degradation is the drug-induced formation of a ternary complex^10,43^. Enabled by the plasticity of a given protein-protein interface, structurally diverse degraders can prompt ternary assemblies of different architectures^2,9^. We hypothesize that, based on the specific geometry of a given assembly, mutations altering the surface topologies of the involved proteins can disrupt the drug-induced molecular proximity, preventing target degradation and ultimately leading to drug resistance. Here, we focus our efforts on CRBN and VHL. In the presented examples, we leverage cytotoxic effects of drugs resulting from degradation of widely essential proteins. Hence, variant selection was based on an altered cellular fitness as a downstream readout for drug-induced target degradation. Noteworthy, the presented DMS approach could also be combined with FACS-based readouts, thus expanding its reach also to non-essential targets or pathways. Based on the resistance-causing mutations we initially identified via targeted re-sequencing in near-haploid human cells, we have focused the mutational scanning on residues that are proximal to the degrader binding site. This focus was chosen to obtain a relatively manageable library size of around 1500 variants each, yet prevented the identification of hotspots outside the dimerization interface.

In general terms, we anticipate that multi-layered maps of functional E3 hotspots can advance our understanding of determinants of drug-induced substrate recognition by E3 ligases. We perceive this approach to be highly complementary and synergistic with efforts in structural biology of degrader ternary complexes. It provides scalable and functional information in the context of a cellular environment involving native protein components. For TPD-compatible E3 ligases lacking structural data, design of variant libraries and mechanistic interpretations will arguably be more challenging ^6^. However, protein structure prediction and ternary complex modeling could offer insights, particularly in cases where the degrader binding site on the E3 could be mapped^44,45^. Additionally, or in absence of interpretable predictions, one could initially scan the entire gene CRISPR-tiling to then dissected functionally relevant interfaces in-depth via DMS.

Intriguingly, some of the identified and validated functional hotspots could not sufficiently be rationalized based on existing structural models. Among others, this is exemplified by functional hotspots that involve the BET PROTAC ARV-771. Based on the presented DMS data, for instance exemplified by VHL^P71I^ and VHL^H110L^, it is conceivable that ARV-771 induces a ternary complex of a different geometry than the ones previously resolved for MZ1^10^ or macroPROTAC-1^34^. In support of these predictions are the observations that (i) ARV-771-induced ternary complex assemblies have thus far proven to be unsuccessful to crystallization efforts; (ii) ARV-771 and MZ1 displayed distinct intra-BET bromodomain cooperativity profiles in FP ternary complex assays^46^. Hence, this and related observations emerging from this study underscore that nuanced, differentiated mutational profiles and sensitivities can arise even with degraders which share otherwise highly similar chemical structures, mechanisms, and cellular activities.

Finally, we hope that our multi-layered maps of functional hotspots in CRBN and VHL will also inform potential resistance mechanisms, as well as ways to overcome them by altered degrader design. In line with previous studies that employed CRISPR/Cas9 screens^13–15^, we show that most emerging mutations occur directly in the SR of the involved E3 ligase. Of note, our sequencing strategy is limited in detecting copy number loss or splicing defects, and hence doesn’t cover the full spectrum of possible causative mutations. Intriguingly, our data highlight that the essentiality of the co-opted SR appears to correlate with the frequency, type and topology of the identified alterations, even though we can’t exclude the contribution of additional factors. While it appears reasonable to conclude that resistance-causing mutations will be enriched in the ligase, mutations can also arise on the neo-substrate, as for instance reported for CDK12-targeting PROTACs^47^. Moreover, an elegant recent study described a complementary approach, which is based on a CRISPR-suppressor scanning strategy, to identify resistance-causing mutations that are localized in neo-substrates of known molecular glue degraders^48^.

Which mutations will turn out to be clinically relevant will only be revealed when additional degraders will be clinically evaluated. As of now, evidence from clinical practice is only available for CRBN-based IMiDs, such as lenalidomide and pomalidomide. Accumulating data has shown that up to one-third of patients refractory to pomalidomide treatment present with various types of CRBN alterations^41,42,49^. In support of a potential clinical relevance of our DMS approach, we found that a number of the identified hotspots are disrupted in patients relapsing from IMiD treatment. Some of the identified hotspots appeared to be specific for molecular glues, such as CRBN^P352^, while others were similarly required for PROTAC potency, for example CRBN^F381^. Of note, our DMS reconstitution mimics the scenario of homozygous mutations, while mutations in patients might also be heterozygous. Future data on clinical trials of CRBN-based glue degraders, such as CC-90009, and CRBN-based PROTACs, such as ARV-471 (targeting the estrogen receptor) and ARV-110 (targeting the androgen receptor) or VHL-based PROTACs, such as DT-2216 (targeting Bcl-xL) will likely shed light on additionally clinically relevant functional hotspots^7^.

## Materials and Methods

### Cell lines, tissue culture and lentiviral transduction

KBM7 cells were obtained from T. Brummelkamp and grown in IMDM supplemented with 10% FBS and 1% penicillin/streptomycin (pen/strep). All other cells were obtained from ATCC or DSMZ. RKO, 293T and HeLa cells were cultured in DMEM supplemented with 10% FBS and 1% pen/strep. MOLM-13 and MV4;11 were grown in RPMI, 10% FBS and 1% pen/strep. pSpCas9(BB)-2A-GFP (PX458) was obtained through Addgene (48138) and used to transiently express sgRNA against CRBN and VHL in RKO cells (see Supplementary Table 4). Clones were single cell seeded and checked for CRBN/VHL deletion via PCR on gDNA or Western blotting. pENTR221_CRBN_WT (a gift from J. Bradner) and pDONR223_VHL_WT (Addgene 81874) were used to generate single CRBN and VHL variants via Q5 site-directed mutagenesis (New England Biolabs, E0554S) and subsequently cloned via Gibson Assembly in the pRRL-EF1a-XhoI-IRES-BlastR plasmid (gift from J. Bigenzahn and G. Superti-Furga) using the NEBuilder HiFi DNA Assembly Mix (New England Biolabs, E2621L). The CRBN/VHL WT and point mutant plasmids were used for lentivirus production and subsequent transduction in RKO CRBN^-/-^ and VHL^-/-^ clones, respectively.

For lentiviral production, 293T cells were seeded in 10 cm dishes and transfected at approx. 80 % confluency with 4 μg target vector, 2 μg pMD2.G (Addgene 12259) and 1 μg psPAX2 (Addgene 12260) using PEI (PolyScience, 24765-100) and following standard protocol. ^51^ Viral supernatant was harvested after 60 h, filtrated and stored in aliquots at - 80 °C for transduction.

### Colony formation assays

Cells were seeded in 6 well plates at a cell density of 1’000 cells/well and treated with DMSO or the indicated drug. After 10 days, cell colonies were stained with Crystal Violet (Cristal Violet 0.05% w/v, Formaldehyde 1%, 1x PBS, Methanol 1%) for 20 min, washed with water and dried. Colony number and density were quantified with ImageJ (US National Institutes of Health, ColonyArea plugin)^52^.

### Cell viability assays

Cells were seeded in 96- well plates at a cell density of 5000 cells per well and treated for 3 or 4 days with DMSO or drug at ten different 1:5 serial diluted concentrations. Starting concentrations of the drugs: ACBI1 20 μM (Boehringer Ingelheim, opnme), ARV-771 1 μM (MedChem Express, HY-100972), MZ1 10 μM, AT7 10 μM, macroPROTAC-1 20 μM, CC-90009 20 μM (MedChem Express, HY-130800), dBET6 1 μM (MedChem Express, HY-112588), dBET57 20 μM (MedChem Express, HY-123844). Each treatment was performed in biological triplicates. Cell viability was assessed via the CellTiter Glo assay according to manufacturer instructions (CellTiter-Glo Luminescent Cell Viability Assay, Promega G7573). Luminescence signal was measured on a Multilabel Plate Reader Platform Victor X3 model 2030 (Perkin Elmer). Survival curves and half-maximum effective concentrations (EC50) were determined in GraphPad Prism version 8.4.2 by fitting a nonlinear regression to the log10 transformed drug concentration and the relative viability after normalization of each data point to the mean luminescence of the lowest drug concentration.

### Western blot analysis

PBS-washed cell pellets were lysed in RIPA Buffer (50 mM Tris-HCl pH 8.0, 150 mM NaCl, 1% Triton X-100, 0.5% sodium deoxycholate, 0.1% SDS, 1× Halt protease inhibitor cocktail, 25 U ml^–1^ Benzonase). Lysates were cleared by centrifugation for 15 min at 4 °C and 20,000*g*. Protein concentration was measured by BCA according to the manufacturer’s protocol (Fisher Scientific Pierce BCA Protein Assay Kit, 23225) and 4X LDS sample buffer was added. Proteins (20 μg) were separated on 4-12% SDS-PAGE gels and transferred to nitrocellulose membranes. Membranes were blocked with 5% milk in TBST for 30 min at RT. Primary antibodies were incubated in milk or TBST alone for 1 h at RT or 4°C overnight. Secondary antibodies were incubated for 1 h at RT. Blots were developed with chemiluminescence films. Primary antibodies used: BRD4 (1:1000, Abcam, ab128874), BRD3 (1:1000, Bethyl Laboratories, A302-368A), BRD2 (1:1000, Bethyl Laboratories, A302-582A), SMARCA4 (1:1000, Bethyl Laboratories, A300-813A), SMARCA2 (1:1000, Cell Signaling Technology, #6889), cMYC (1:1000, Santa Cruz Biotechnology, sc-764), GSPT1 (1:1000, Abcam, ab49878), CDK9 (1:1000, Cell Signaling Technology, 2316S), CRBN (1:2000, kind gift of R. Eichner and F. Bassermann), VHL (1:1000, Cell Signaling Technology, 2738), ACTIN (1:5000, Sigma-Aldrich, A5441-.2ML), GAPDH (1:1000, Santa Cruz Biotechnology, sc-365062). Secondary antibodies used: Peroxidase-conjugated AffiniPure Goat Anti-Rabbit IgG (1:10000, Jackson ImmunoResearch, 111-035-003) and Peroxidase-conjugated AffiniPure Goat Anti-Mouse IgG (1:10000, Jackson ImmunoResearch, 115-035-003).

### Resistance rate determination

KBM7 cells (4 x 10^6^) were treated at a single dose relative to the degraders EC_50_ values in 3-day dose response assays (see also Extended Data Fig. 1A) in 20 ml of media. Cells were then seeded into 384-well plates at 50 μl per well and after 21 days, wells with proliferating cells were counted for each treatment. To correct for wells containing more than one resistant cell, the probability *p* of obtaining resistant cells was calculated via a binomial distribution using the count of wells lacking resistant cells according to the following formula, where n is 10000 (cells per well) and P(x = 0) is the fraction of non-outgrowing wells on the plate.

$$P(x=0)=\left(\frac{n}{x}\right){\left(1-p\right)}^{n}$$

### Acquired resistance mutation identification by hybrid capture

#### Generation of acquired drug resistant cells and hybrid-capture library preparation for next-generation sequencing

One hundred million KBM7 cells were treated with DMSO or 10X (100 nM), 25X (250 nM), 50X (500 nM) EC_50_ of dBET6 or ARV in 50 ml medium. After 25 d, Ficoll-gradient centrifugation with Lymphocyte Separation Media (Corning, COR25-072-CV) was performed according to manufacturer’s protocols. Cells were recovered for one day, counted and PBS washed pellets were stored at -80 °C for subsequent gDNA extraction (QIAamp DNA Mini, QIAGEN 51304). DNA content was determined with Qubit dsDNA HS Kit (Thermo Fisher, Q32854) and 500 ng of the gDNA was subjected to DNA library preparation using the NEBNext Ultra II FS DNA Library Prep kit for Illumina (New England Biolabs, E7805S) following manufacturer’s instructions (protocol for inputs >100 ng). Fragments were size-selected using AMPure XP beads (Beckman Coulter, 10136224) for fragments of 150-350 bp. Adaptor-ligated DNA was amplified in five cycles by PCR using NEBnext Multiplex Oligos for Illumina (Set1 E7335 and Set2 E75000). For hybrid capture, xGen Gene Capture Pools for the 29 genes of interest were purchased from IDT (see Supplementary Table 1) and 500 ng of DNA was used as input. Hybridization was performed for 16h following the supplier’s protocols, including the xGen Universal Blocker-TS Mix (IDT, 1075475) blocking oligos. Post-capture PCR was performed with the NEBNext High-Fidelity 2X PCR Master Mix (NEB, M0541S) for 14-20 cycles. Sequencing libraries were quantified using the Qubit dsDNA HS Kit (Thermo Fisher Q32854) and analyzed on an Agilent 2100 Bioanalyzer before sequencing on a HiSeq 4000 lane (50 bp single-end).

#### NGS data analysis

Raw sequencing reads were converted to fastq files using the bamtools convert (v2.5.1)^53^. Sequencing adapters and low-quality reads were trimmed using the Trimmomatic tool (v0.39) in SE mode with standard settings^54^. Reads were aligned to the hg38/GRCh38 assembly of the human reference genome using aln and samse algorithms from the bwa package (v0.7.17)^55^. Unmapped reads were removed using the CleanSam function from the Picard toolkit (v2.25.1, Broad Institute GitHub Repository). Reads were sorted and duplicate reads filtered using the SortSam and MarkDuplicates Picard tools. Read groups were added by the Picard AddOrReplaceReadGroups tool.

The Mutect2 function from the GATK (v4.1.8.1) was used to call variants. The variants were annotated using the Ensembl Variant Effect Predictor tool (v103.1)^56^. Coding variants with greater than 2-fold enrichment in allele frequency (as determined by Mutect2) upon drug treatment compared to the wild-type population were considered hits (see also Supplementary Dataset).

### Deep mutational scanning screens

#### Design, cloning and lentiviral production of the DMS library

Amino acid residues within 10 Å of the VHL-ligand 1 and thalidomide binding pockets on VHL and CRBN respectively were determined via PyMol (v2.3.5) and selected for site saturation library design by TWIST Biosciences. Pooled libraries of mutant VHL (1442 variants) and CRBN (1738 variants) were introduced into the XhoI digested backbone pRRL-EF1a-XhoI-IRES-BlastR with NEBuilder 2x HiFi assembly (New England Biolabs). The assembly mix was purified via isopropanol precipitation and electroporated into Stbl4 bacteria (Thermo Fisher, 11635018) at 1.2 kV, 25 μF and 200 Ω. After recovery, the bacterial suspension was plated on LB Agar plates containing Ampicillin for selection. Dilutions of the bacterial suspension were plated and counted to determine a library coverage of 135x and 54x for VHL and CRBN libraries respectively. Quality control of the library distribution was performed via next-generation sequencing of the plasmid preparation as outlined for the screens below, except that the mentioned PCR was performed for 5 cycles. 1442 of 1500 possible VHL variants and 1738 of 1740 CRBN substitutions were recovered in the libraries. The VHL library included an abundant mutant (F119I) caused by library synthesis, which had no functional inconsequence. Lentiviral supernatant was produced as mentioned earlier and concentrated using Lenti-X concentrator (Takara, 631232) followed by storage at -80°C in aliquots.

#### Deep mutational scanning library screens

Eight million RKO CRBN^-/-^ or VHL^-/-^ were transduced at a MOI of 0.3 yielding a calculated library representation of 1664 and 1380 cells per variant for VHL and CRBN respectively. For each transduction one million cells were seeded in a 12-well plate with 8 μgml^-1^ polybrene (SantaCruz, SC-134220), the titrated amount of lentivirus filled to 1 ml with culture media. The plate was centrifuged at 765 x g for 1 h at 37°C and cells were detached after 6 h of incubation at 37°C, pooled and expanded. 48 hrs after transduction, pools were selected by adding 20 μgml^-1^ blasticidine for 7 days. Independent mutational scanning resistance screens were performed in replicates by treating 2.5 million cells, splitting and retreating after 4 days and harvesting 2.5 million cell pellets after a total of 7 day treatment with the indicated drug and dose.

#### Library preparation for next-generation sequencing

Genomic DNA (gDNA) was extracted from frozen cell pellets following the QIAamp DNA Mini Kit (Qiagen, 51304). VHL and CRBN variant cDNAs were amplified via PCR from gDNA with primers CRBN_GA_fwd & rev and VHL_GA_fwd & rev respectively. Primer sequences are available in Supplementary Table 4. The total isolated gDNA was processed in batches of 5 μg per PCR reaction with Q5 polymerase (NEB, M0491L). One PCR reaction contained 10 μl 5x reaction buffer, 10 μl 5x GC enhancer, 2.5 μl primer mix containing 10 μM forward and reverse primer each, 1 μl dNTP mix (10 μM each), 1 μl Q5 polymerase and nuclease-free water to bring the reaction volume to 50 μl. Target amplification was achieved by performing: 30 s initial denaturation at 95°C; next for 20 to 28 cycles: 15 s at 95°C, 30 s at 57°C and 2 min at 72°C; followed by a final extension for 5 min at 72°C. The cycle number for specific amplification of the 700 base-pair (VHL) and 1.4 kilo-base-pair (CRBN) targets was confirmed by agarose gel electrophoresis. PCR reactions for each treatment were pooled and purified using AMPure XP beads (Beckman Coulter, 10136224) according to standard protocol for double-sided clean up in a 0.3:1 and 1:1 ratio. The purity and integrity of the PCR products were analysed on an Agilent 2100 Bioanalyzer following manufacturer recommendations for high sensitivity DNA chips (Agilent, 5067-4626). Sequencing libraries were prepared using Nextera DNA Library Prep Kit (Illumina, FC-131-1024) following standard manufacturer instructions for amplicon libraries. This cuts the PCR products and tags resulting pieces with adapter sequences for the following sequencing. After purification of the fragmented and PCR amplified DNA libraries, quality control was performed by analysis on an Agilent 2100 Bioanalyzer following manufacturer recommendations for high sensitivity DNA chips (Agilent, 5067-4626). Final sequencing libraries were pooled in equimolar amounts and sequenced running 50-bp single-end reads on a HiSeq4000.

#### NGS data analysis

Raw sequencing reads were converted to fastq format using samtools (v1.10). Sequencing adapters were removed, and low-quality reads were filtered using the Trimmomatic tool (v0.39) in SE mode with standard settings^54^. Short reads were aligned to the expression cassette using aln algorithm from the bwa software package (v0.7.17) with the -n 5 parameter allowing for 5 mismatches, followed by bwa samse command to generate SAM files^55^. Alignment files were sorted using SortSam function from the Picard toolkit (v2.25.1, Broad Institute GitHub Repository). Mutation calling was performed using the AnalyzeSaturationMutagenesis tool from GATK (v4.1.8.1)^57^. Given our sequencing strategy, 98.89 % of reads constituted wild type sequences and were therefore filtered out during this step. Next, relative frequencies of variants were calculated for each interrogated position and variants that were covered by less than 1 in 10,000 reads in the DMSO sample were excluded from further quantitative analysis. Read counts for each variant were then normalized to total read count of each sample and log2FCs of treatment over DMSO were calculated. To correct for differential drug potency, we next normalized each variant to the maximum log2 fold-change over DMSO. For drug comparisons, log2 fold-changes over DMSO were subtracted. Given the sequencing of 50-bp reads, cDNAs harbouring two mutations (from synthesis errors) in greater distance will not be detected as multiple mutations with this strategy and hence present as 2 separate variants. Heatmaps were generated using pheatmap (v1.0.12) package in R (v4.1.2). Mapping of median resistance scores per residue on protein structures was performed using the PyMOL software (v2.5.2, Schrödinger LLC) using publicly available protein structures of CRBN (PDB: 6BOY) and VHL (PDB: 4W9H).

### Competition growth experiments

KBM7 cells constitutively expressing Cas9_Blast (Addgene #52962) were transduced with lentivirus expressing sgRNAs against *VHL*, *GAPDH*, *RPL5* or in the gene desert of *MYC* in the GFP vector LRG (Lenti_sgRNA_EFS_GFP) (Addgene #65656, see Supplementary Table 4). GFP-expressing cells were mixed with GFP-negative cells at a 1:1 ratio. The mixed populations were grown for 21 days, and monitored by flow cytometry in 7-day intervals. Data was analyzed with FlowJo (gating strategy see Supplementary Figure 3) and percentages of the respective GFP populations were normalized to day 0.

### Recombinant protein generation

Protein production for SMARCA4, BRD4.2 and the WT VCB complex was carried out as previously described^10,35^. The VCB mutants, in which R67 and P71I of VHL (54-213) were mutated to glutamine and isoleucine respectively, were generated using a Q5 site directed mutagenesis kit (NEB, E0554S) according to the manufacturer’s instructions and expressed and purified as for VCB. Mass spectrometry analysis and agarose gel electrophoresis was carried out to ensure purity of the recombinant proteins (see Supplementary Figure 3).

### Fluorescence polarization

FP competitive binding assays were performed as described previously^58^, with all measurements taken using a PHERAstar FS (BMG LABTECH) with fluorescence excitation and emission wavelengths (λ) of 485 and 520 nm, respectively. Assays were run in triplicate using 384-well plates (Corning, 3544), with each well solution containing 15 nM VCB protein, 10 nM 5,6-carboxyfluorescein (FAM)-labeled HIF-1α peptide (FAM-DEALAHypYIPMDDDFQLRSF, “JC9”), and decreasing concentrations of PROTACs (11-point, 3-fold serial dilution starting from 40 μM) or PROTACs:bromodomain (11-point, 3-fold serial dilution starting from 40 μM PROTAC: 80 μM bromodomain into buffer containing 40 μM of bromodomain). All components were dissolved from stock solutions using 100 mM Bis–Tris propane, 100 mM NaCl, 1 mM DTT, pH 7.0, to yield a final assay volume of 15 μL. DMSO was added as appropriate to ensure a final concentration of 2% v/v. Control wells containing VCB and JC9 with no compound or JC9 in the absence of protein were also included to allow for normalization. IC_50_ values were determined for each titration using nonlinear regression analysis with Prism (GraphPad). Cooperativity values (α) for each PROTAC were calculated using the ratio: α = IC_50_ (− bromodomain)/ IC_50_ (+ bromodomain).

### Crystallography

The ternary complex VCB: AT7:Brd4^BD2^ was prepared by combining VCB, Brd4^BD2^, and AT7 in a 1:1:1 molar ratio and incubating for 15 min at RT. Crystals were grown at 20 °C using the hanging drop diffusion method by mixing equal volumes of ternary complex solution and a crystallization solution containing 10% (w/v) PEG 8000, 0.1 M Tris-HCl (pH 7.5) and 0.1 M MgCl_2_. Crystals were ready for harvest within 24 h and were flash-frozen in liquid nitrogen using 20% (v/v) ethylene glycol in liquor solution as a cryoprotectant. Diffraction data were collected at Diamond Light Source beamline I24 using a Pilatus 6M-F detector at a wavelength of 0.9750 Å. Reflections were indexed and integrated using XDS, and scaling and merging were performed with AIMLESS in CCP4i (v7.1.018)^59^. The crystals belonged to space group P_32_, with two copies of the ternary complex in the asymmetric unit. The structure was solved by molecular replacement using MOLREP and search models derived from the coordinates for the VCB:MZ1:Brd4^BD2^ ternary complex (PDB entry 5T35). The initial model underwent iterative rounds of model building and refinement with COOT and REFMAC5, respectively. All riding hydrogens were excluded from the output coordinate files but included for refinement. Compound geometry restraints for refinement were prepared with the PRODRG server. Model geometry and steric clashes were validated using the MOLPROBITY server.^60^ The structure has been deposited in the protein data bank (PDB: 7ZNT); data collection and refinement statistics are presented in Supplementary Table 3. Interfaces observed in the crystal structure were calculated using PISA, and all figures were generated using PyMOL.

## Extended Data

## Supplementary Material

Supplementary dataset

Supplementary Information

## Data Availability

Raw and analysed mutational scanning and hybrid capture datasets (Figures 1 to 5, S1 and S3 to 5) are available in the Gene Expression Omnibus database under accession code GSE198280. For their analysis the human reference genome (hg38/GRCh38 assembly, GenBank ID 883148) was used. Atomic coordinates and structure factors for the new protein structure VCB:AT7:Brd4^BD2^ is available at the protein data bank (PDB: 7ZNT). All data generated and analysed in this study are included in this published article, its Supplementary Information, the mentioned databases or are available from the corresponding authors upon request.

## Code availability

All code used for analysis of the experimental data is available at https://github.com/GWinterLab/TPDR.

## References

[1] Deshaies RJ (2020). Multispecific drugs herald a new era of biopharmaceutical innovation. Nature.

[2] Gerry CJ, Schreiber SL (2020). Unifying principles of bifunctional, proximity-inducing small molecules. Nat Chem Biol.

[3] Hanzl A, Winter GE (2020). Targeted protein degradation: current and future challenges. Curr Opin Chem Biol.

[4] Petroski MD, Deshaies RJ (2005). Function and regulation of cullin-RING ubiquitin ligases. Nat Rev Mol Cell Biol.

[5] Harper JW, Schulman BA (2021). Cullin-RING Ubiquitin Ligase Regulatory Circuits: A Quarter Century Beyond the F-Box Hypothesis. Annu Rev Biochem.

[6] Kramer LT, Zhang X (2022). Expanding the landscape of E3 ligases for targeted protein degradation. Current Research in Chemical Biology.

[7] Békés M, Langley DR, Crews CM (2022). PROTAC targeted protein degraders: the past is prologue. Nat Rev Drug Discov.

[8] Kozicka Z, Thomä NH (2021). Haven’t got a glue: Protein surface variation for the design of molecular glue degraders. Cell Chem Biol.

[9] Maniaci C, Ciulli A (2019). Bifunctional chemical probes inducing protein-protein interactions. Curr Opin Chem Biol.

[10] Gadd MS (2017). Structural basis of PROTAC cooperative recognition for selective protein degradation. Nat Chem Biol.

[11] Nowak RP (2018). Plasticity in binding confers selectivity in ligand-induced protein degradation. Nat Chem Biol.

[12] Zorba A (2018). Delineating the role of cooperativity in the design of potent PROTACs for BTK. Proc Natl Acad Sci U S A.

[13] Shirasaki R (2021). Functional Genomics Identify Distinct and Overlapping Genes Mediating Resistance to Different Classes of Heterobifunctional Degraders of Oncoproteins. Cell Rep.

[14] Sievers QL, Gasser JA, Cowley GS, Fischer ES, Ebert BL (2018). Genome-wide screen identifies cullin-RING ligase machinery required for lenalidomide-dependent CRL4(CRBN) activity. Blood.

[15] Mayor-Ruiz C (2019). Plasticity of the Cullin-RING Ligase Repertoire Shapes Sensitivity to Ligand-Induced Protein Degradation. Mol Cell.

[16] Zhang L, Riley-Gillis B, Vijay P, Shen Y (2019). Acquired Resistance to BET-PROTACs (Proteolysis-Targeting Chimeras) Caused by Genomic Alterations in Core Components of E3 Ligase Complexes. Mol Cancer Ther.

[17] Ferguson FM, Gray NS (2018). Kinase inhibitors: the road ahead. Nat Rev Drug Discov.

[18] Zaidman D, Prilusky J, London N (2020). ProsetTac: Rosetta based modeling of PROTAC mediated ternary complexes. J Chem Inf Model.

[19] Bai N (2021). Rationalizing PROTAC-Mediated Ternary Complex Formation Using Rosetta. J Chem Inf Model.

[20] Drummond ML, Williams CI (2019). In Silico Modeling of PROTAC-Mediated Ternary Complexes: Validation and Application. J Chem Inf Model.

[21] Sievers QL (2018). Defining the human C2H2 zinc finger degrome targeted by thalidomide analogs through CRBN. Science.

[22] Eron SJ (2021). Structural Characterization of Degrader-Induced Ternary Complexes Using Hydrogen-Deuterium Exchange Mass Spectrometry and Computational Modeling: Implications for Structure-Based Design. ACS Chem Biol.

[23] Dixon T (2021). Atomic-Resolution Prediction of Degrader-mediated Ternary Complex Structures by Combining Molecular Simulations with Hydrogen Deuterium Exchange. bioRxiv.

[24] Meyers RM (2017). Computational correction of copy number effect improves specificity of CRISPR-Cas9 essentiality screens in cancer cells. Nat Genet.

[25] Latif F (1993). Identification of the von Hippel-Lindau disease tumor suppressor gene. Science.

[26] Raina K (2016). PROTAC-induced BET protein degradation as a therapy for castration-resistant prostate cancer. Proc Natl Acad Sci U S A.

[27] Winter GE (2017). BET Bromodomain Proteins Function as Master Transcription Elongation Factors Independent of CDK9 Recruitment. Mol Cell.

[28] Forment Jv (2017). Genome-wide genetic screening with chemically mutagenized haploid embryonic stem cells. Nat Chem Biol.

[29] Volz JC, Schuller N, Elling U (2019). Using Functional Genetics in Haploid Cells for Drug Target Identification. Methods Mol Biol.

[30] Winter GE (2014). The solute carrier SLC35F2 enables YM155-mediated DNA damage toxicity. Nat Chem Biol.

[31] Suiter CC (2020). Massively parallel variant characterization identifies NUDT15 alleles associated with thiopurine toxicity. Proc Natl Acad Sci U S A.

[32] Awad MM (2021). Acquired Resistance to KRAS G12C Inhibition in Cancer. N Engl J Med.

[33] Zengerle M, Chan KH, Ciulli A (2015). Selective Small Molecule Induced Degradation of the BET Bromodomain Protein BRD4. ACS Chem Biol.

[34] Testa A, Hughes SJ, Lucas X, Wright JE, Ciulli A (2020). Structure-Based Design of a Macrocyclic PROTAC. Angew Chem Int Ed Engl.

[35] Farnaby W (2019). BAF complex vulnerabilities in cancer demonstrated via structure-based PROTAC design. Nat Chem Biol.

[36] Soares P (2018). Group-Based Optimization of Potent and Cell-Active Inhibitors of the von Hippel-Lindau (VHL) E3 Ubiquitin Ligase: Structure-Activity Relationships Leading to the Chemical Probe (2S,4R)-1-((S)-2-(1-Cyanocyclopropanecarboxamido)-3,3-dimethylbutanoyl)-4-hydroxy-N-(4-(4-methylthiazol-5-yl)benzyl)pyrrolidine-2-carboxamide (VH298). J Med Chem.

[37] Matyskiela ME (2016). A novel cereblon modulator recruits GSPT1 to the CRL4(CRBN) ubiquitin ligase. Nature.

[38] Surka C (2021). CC-90009, a novel cereblon E3 ligase modulator, targets acute myeloid leukemia blasts and leukemia stem cells. Blood.

[39] Fink EC (2018). Crbn I391V is sufficient to confer in vivo sensitivity to thalidomide and its derivatives in mice. Blood.

[40] Olson CM (2017). Pharmacological perturbation of CDK9 using selective CDK9 inhibition or degradation. Nat Chem Biol.

[41] Barrio S (2020). IKZF1/3 and CRL4 CRBN E3 ubiquitin ligase mutations and resistance to immunomodulatory drugs in multiple myeloma. Haematologica.

[42] Gooding S (2021). Multiple cereblon genetic changes are associated with acquired resistance to lenalidomide or pomalidomide in multiple myeloma. Blood.

[43] Roy MJ (2019). SPR-Measured Dissociation Kinetics of PROTAC Ternary Complexes Influence Target Degradation Rate. ACS Chem Biol.

[44] Jumper J (2021). Highly accurate protein structure prediction with AlphaFold. Nature.

[45] Scholes NS, Mayor-Ruiz C, Winter GE (2021). Identification and selectivity profiling of small-molecule degraders via multi-omics approaches. Cell Chem Biol.

[46] Klein VG, Bond AG, Craigon C, Lokey RS, Ciulli A (2021). Amide-to-Ester Substitution as a Strategy for Optimizing PROTAC Permeability and Cellular Activity. J Med Chem.

[47] Jiang B (2021). Discovery and resistance mechanism of a selective CDK12 degrader. Nat Chem Biol.

[48] Gosavi PM (2022). Profiling the Landscape of Drug Resistance Mutations in Neosubstrates to Molecular Glue Degraders. ACS Cent Sci acscentsci.

[49] Kortum KM (2016). Targeted sequencing of refractory myeloma reveals a high incidence of mutations in CRBN and Ras pathway genes. Blood.

[50] Galdeano C (2014). Structure-guided design and optimization of small molecules targeting the protein-protein interaction between the von Hippel-Lindau (VHL) E3 ubiquitin ligase and the hypoxia inducible factor (HIF) alpha subunit with in vitro nanomolar affinities. J Med Chem.

[51] Joung J (2017). Genome-scale CRISPR-Cas9 knockout and transcriptional activation screening. Nat Protoc.

[52] Guzmán C, Bagga M, Kaur A, Westermarck J, Abankwa D (2014). ColonyArea: an ImageJ plugin to automatically quantify colony formation in clonogenic assays. PLoS One.

[53] Barnett D, Garrison EK, Quinlan AR, Strmberg MP, Marth G, BamTools T (2011). a C API and toolkit for analyzing and managing BAM files. Bioinformatics.

[54] Bolger AM, Lohse M, Usadel B (2014). Trimmomatic: a flexible trimmer for Illumina sequence data. Bioinformatics.

[55] Li H, Durbin R (2009). Fast and accurate short read alignment with Burrows-Wheeler transform. Bioinformatics.

[56] McLaren W (2016). The Ensembl Variant Effect Predictor. Genome Biol.

[57] McKenna A (2010). The Genome Analysis Toolkit: a MapReduce framework for analyzing next-generation DNA sequencing data. Genome Res.

[58] van Molle I (2012). Dissecting Fragment-Based Lead Discovery at the von Hippel-Lindau Protein:Hypoxia Inducible Factor 1α Protein-Protein Interface. Chem Biol.

[59] Potterton E, Briggs P, Turkenburg M, Dodson E (2003). A graphical user interface to the CCP4 program suite. Acta Crystallogr D Biol Crystallogr.

[60] Chen VB (2010). MolProbity: all-atom structure validation for macromolecular crystallography. Acta Crystallogr D Biol Crystallogr.

