# Supplementary material for: Functional E3 ligase hotspots and resistance mechanisms to small-molecule degraders
Source: Nat Chem Biol. Author manuscript; Available in PMC 2023 Mar 1. (PMC7614256; doi:10.1038/s41589-022-01177-2)
Supplement: Supplementary Information [file EMS156571-supplement-Supplementary_Informationt.pdf]

Table of Contents:

Supplementary Table 1: List of genes included in xGen Gene Capture Pool
Supplementary Table 2: Degraders used in this manuscript
Supplementary Table 3: Crystallographic data collection and refinement statistics.
Supplementary Table 4. Oligo sequences
Supplementary Figure 1: VHL deep mutational scanning results
Supplementary Figure 2: CRBN deep mutational scanning results
Supplementary Figure 3: Gating Strategy and Characterization of Recombinant Protein
Supplementary Note: Chemical synthesis of AT7

Supplementary Table 1:

List of genes included in xGen Gene Capture Pool

Gene	Function	Median DepMap CRONOS score
BRD2	target	-0.34
BRD3	target	0.11
BRD4	target	-1.05
CAND1	SR exchange	-0.32
CAND2	SR exchange	0.03
COPS2	de-neddylation	-1.15
COPS3	de-neddylation	-0.88
COPS4	de-neddylation	-0.98
COPS5	de-neddylation	-1.61
COPS6	de-neddylation	-1.50
COPS7A	de-neddylation	-0.03
COPS7B	de-neddylation	0.05
COPS8	de-neddylation	-1.11
COPS8	de-neddylation	-1.11
COPS9	de-neddylation	-0.14
CRBN	CRL4 subunit	0.00
CUL2	CRL2	-0.51
CUL4A	CRL4	-0.03
CUL4B	CRL4	-0.01
DDB1	CRL4 subunit	-1.95
ELOB	CRL2 subunit	-1.46
ELOC	CRL2 subunit	-1.17
GPS	de-neddylation	-0.69
NAE1	neddylation	-1.31
RBX1	CRL subunit	-1.34
UBA3	neddylation	-0.93
UBE2F	neddylation	-0.01
UBE2G1	E2 enzyme	-0.03
UBE2M	neddylation	-1.21
UBE2R2	E2 enzyme	-0.04
VHL	CRL2 subunit	-0.96

Supplementary Table 2: Degraders applied in this study

Degrader	Structure	Targets	ref.
**ARV-771**	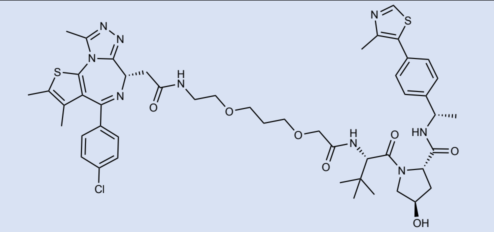	BRD4, BRD3, BRD2	ref. 26
**MZ-1**	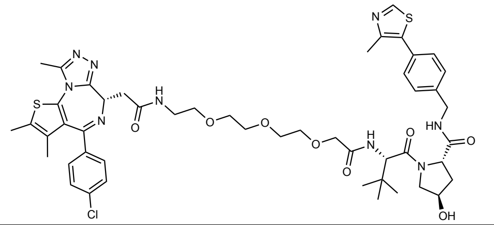	BRD4, BRD3, BRD2	ref. 33
**cis-MZ-1**	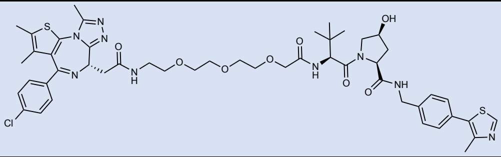	non VHL interacting inhibiting BRD4, BRD3, BRD2	ref. 33
**macroPROTAC-1**	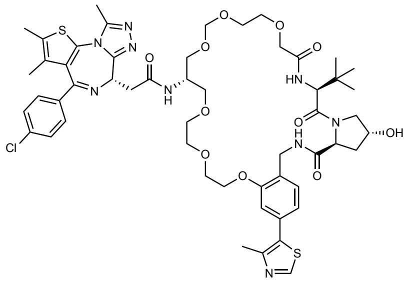	BRD4, BRD3, BRD2	ref. 34
**AT7**	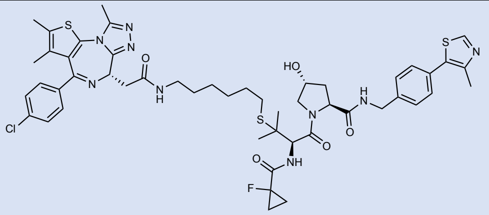	BRD4, BRD3, BRD2	this publication
**ACBI-1**	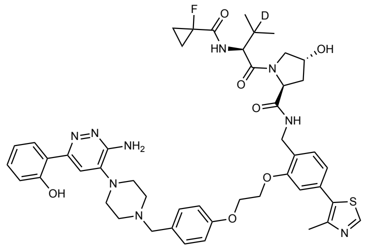	SMARCA2, SMARCA4, PBRM1	ref. 35
**dBET6**	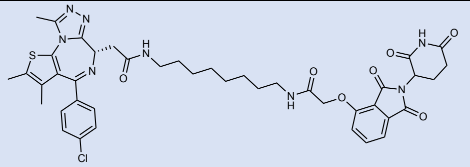	BRD4, BRD3, BRD2	ref. 27
**dBET57**	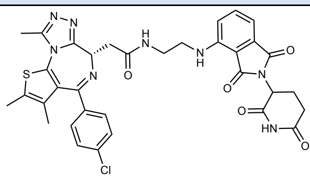	BRD4, BRD3, BRD2	ref. 11
**CC-885**	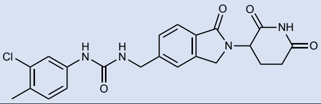	GSPT1	ref. 37
**CC-90009 (Eragidomide)**	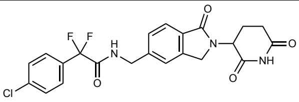	GSPT1	ref. 38

Supplementary Table 3: Crystallographic data collection and refinement statistics.

**Data Collection**	
Space Group	$P3_2$
Cell Dimensions	
a, b, c (Å)	82.6, 82.6, 169.6
α, β, γ, (°)	90.0, 90.0, 120.0
Resolution (Å)	65.9 – 3.0 (3.2 – 3.0)*
No. unique reflections	25970 (4234)
R_{merge} (%)	23.1 (96.6)
I/σ (I)	9.4 (5.3)
$CC_{1/2}$	99.2 (71.3)
Completeness (%)	100.0 (100.0)
Redundancy	9.9 (10.2)
**Refinement**	
R_{work}/R_{free} (%)	21.3/25.1
R.m.s. deviations	
Bond lengths (Å)	0.007
Bond angles (°)	1.363

* Values in parentheses are for highest-resolution shell.

Supplementary Table 4: Oligo sequences

sgRNAs cloned into the plasmid pSpCas9(BB)-2A-GFP Addgene (48138)	
**Gene Name**	**Sequence**
CRBN	TGTATGTGATGTCGGCAGAC
VHL	GCGATTGCAGAAGATGACCT

sgRNAs cloned into the plasmid Lenti_sgRNA_EFS_GFP (Addgene #65656)	
**Gene Name**	**Sequence**
VHL (766)	CGCCGCATCCACAGCTACCG
VHL (767)	AGAGATGCAGGGACACACGA
GAPDH	GATCCCTCCAAAATCAAGTG
RPL5	GATCTATGAAGGCCAAGTGG

Oligos for amplification of CRBN and VHL for DMS	
**Primer Name**	**Sequence**
CRBN_GA fwd	aggtgtcgtgacgtacgggatcccaggaccATGGCCGGCGAAGGAG
CRBN_GA rev	ggggggggggcggaattaattcctactacTTACAAGCAAAGTATTACTTTGTCTGGAC
VHL_GA fwd	aggtgtcgtgacgtacgggatcccaggaccatgccccggagggcggag
VHL_GA rev	ggggggggggcggaattaattcctactactcaatctcccatccgttgatgtgcaatgcg

Supplementary Figure 1: VHL deep mutational scanning results

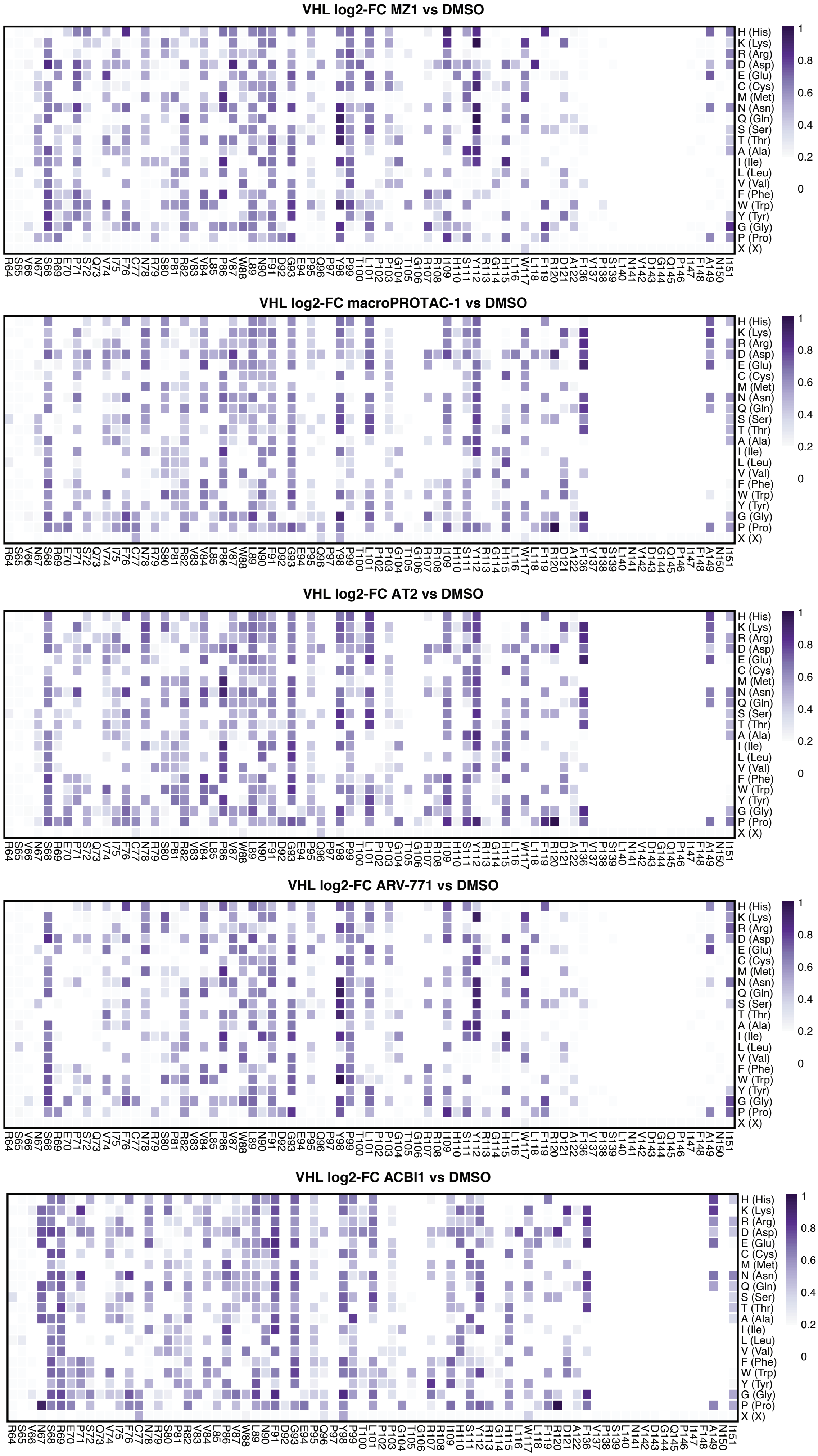

Supplementary Figure 2: CRBN deep mutational scanning results

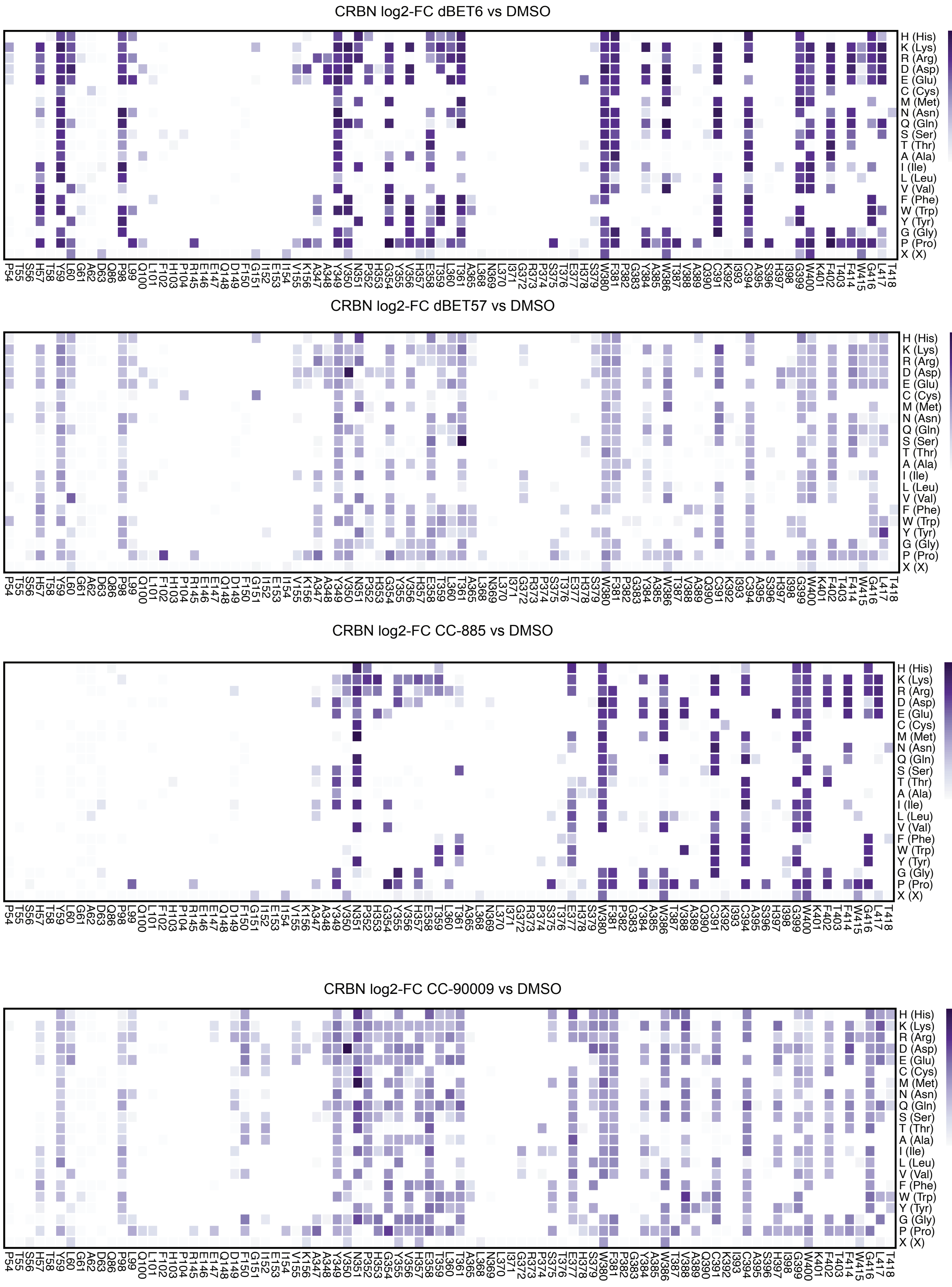

Supplementary Figure 3

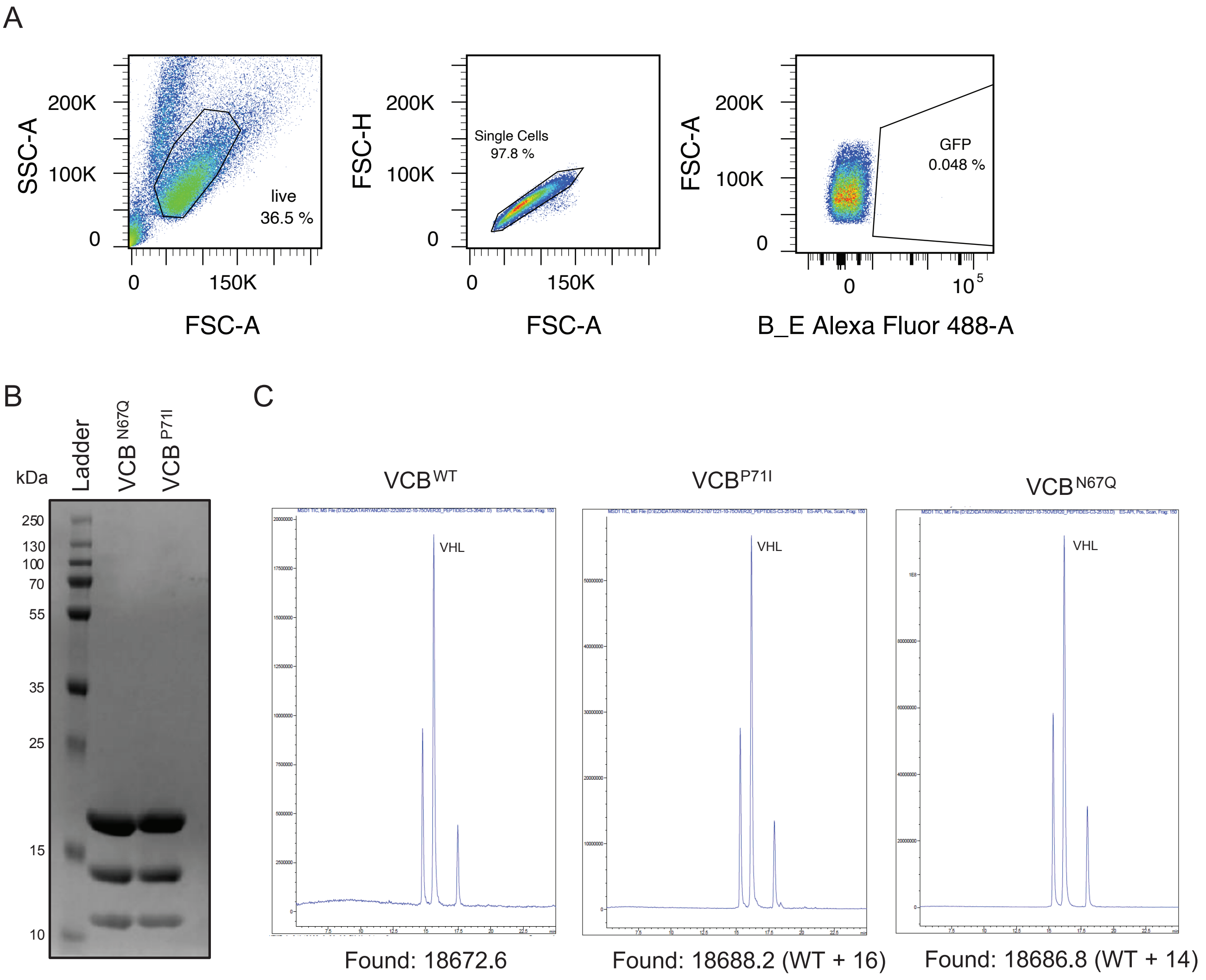

Supplementary Figure 3: Gating Strategy and Characterization of Recombinant Protein
(A) Gating strategy for evaluating the percentage of GFP positive cells related to Extended Data Fig 1B. Shown are untransduced control cells for setting the GFP gate.
(B) Analysis of mutant protein purity and identity following recombinant expression and purification. NuPAGE gel (12% Bis-Tris) of final purified protein sample following staining with InstantBlue with V*BC components running at the expected molecular weights at high purity.
(C) Mass spectrometry chromatogram of final protein samples following separation by HPLC on a C3 column using a gradient of 10 to 75 % acetonitrile over 20 minutes. Data was analysed using an Agilent 6130 quadrupole MS and deconvoluted using Agilent LC/MSD ChemStation and the correct mass shift was identified for each smutant protein when compared to wild-type.

Supplementary Note

Chemical synthesis of AT7

(2S,4R)-1-((R)-2-(1-fluorocyclopropane-1-carboxamido)-3-methyl-3-(tritylthio)butanoyl)-4-hydroxy-N-(4-(4-methylthiazol-5-yl)benzyl)pyrrolidine-2-carboxamide (3)

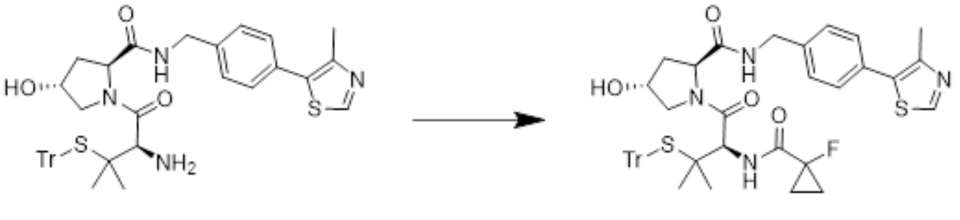

2 **3**

To a solution of **2** (ref. 10) (48 mg, 0.068 mmol) in DMF (0.5 mL) at room temperature, DIPEA (30 µL, 0.172 mmol), HOAT (9mg, 0.068), HATU (26mg, 0.068) and 1-fluorocyclopropanecarboxylic acid (7mg, 0.068 mmol) were added. The mixture was let to react at room temperature for 2 hours. The reaction mixture was cooled to room temperature, filtered and purified by preparative HPLC to give the product (40 mg, 83% yield). MS analysis: $C_{44}H_{45}FN_4O_4S_2$ expected 776.3, found 777.5 [M+H$^+$].

^{1}H NMR (400 MHz, CDCl3) d 8.71 (s, 1H), 7.55 - 7.52 (m, 6H), 7.34 - 7.31 (m, 3H), 7.24 - 7.19 (m, 12H), 4.66 (t, J=8.1 Hz, 1H), 4.37 (br s, 1H), 4.32 - 4.19 (m, 2H), 3.65 (d, J=5.2 Hz, 1H), 3.51 (d, J=11.6 Hz, 1H), 3.26 (dd, J=3.8, 11.6 Hz, 1H), 3.09 (d, J=6.0 Hz, 1H), 2.52 (s, 3H), 2.41 - 2.33 (m, 1H), 2.14 - 2.07 (m, 1H), 1.38 - 1.23 (m, 4H), 1.21 (s, 3H), 0.98 (s, 3H).

^{13}C-NMR (101 MHz, CDCl3, 25 °C) δ: 170.6, 170.4 ($J_{C\text{–}F}$ = 20 Hz), 170.0, 150.4, 148.6, 144.4, 138.2, 131.8, 130.9, 129.9, 129.6, 128.04, 127.99, 127.0, 77.4 ($J_{C\text{–}F}$ = 207 Hz), 70.2, 68.5, 58.5, 57.0, 56.6, 53.7, 43.0, 36.4, 26.1, 25.7, 16.3, 13.9 ($J_{C\text{–}F}$ = 10 Hz), 13.7 ($J_{C\text{–}F}$ = 10 Hz).

(*2S,4R*)-1-((*R*)-2-acetamido-3-mercapto-3-methylbutanoyl)-4-hydroxy-*N*-(4-(4-methylthiazol-5-yl)benzyl)pyrrolidine-2-carboxamide (4)

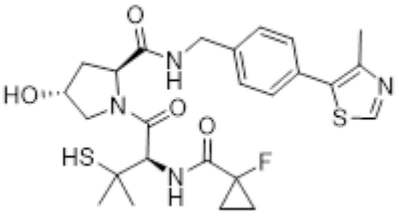

4

Compound **3** (40 mg, 0.057 mmol) was dissolved in 2 mL of DCM. TIPS (0.2 mL) and TFA (0.2 mL) were added, and the yellow mixture was let to react at room temperature for one hour after which LCMS showed complete conversion of the starting material. Volatiles were removed under vacuum and the crude was purified by FCC (from 0 to 15 % of MeOH in DCM) to afford the title compound **4** as a white solid (24 mg, 80% yield). MS analysis: $C_{25}H_{31}FN_4O_4S_2$ expected 534.2, found 535.3 [M+H$^+$].

^{1}H NMR (500 MHz, MeOD) d 8.91 (s, 1H), 8.74 (t, J=5.2 Hz, 1H), 7.72 (d, J=8.3 Hz, 1H), 7.46 (d, J=8.1 Hz, 2H), 7.42 (d, J=7.9 Hz, 2H), 4.93 (d, J=9.0 Hz, 1H), 4.59 (t, J=8.3 Hz, 1H), 4.56 - 4.49 (m, 2H), 4.36 (dd, J=4.8, 15.4 Hz, 1H), 3.96 - 3.85 (m, 2H), 2.47 (s, 3H), 2.25 (dd, J=7.8, 13.1 Hz, 1H), 2.12 - 2.06 (m, 1H), 1.46 (s, 3H), 1.43 - 1.27 (m, 8H).

^{13}C-NMR (101 MHz, CDCl3, 25 °C) δ: 174.2, 171.6 (J_{C-F} = 20 Hz), 170.7, 153.0, 148.7, 140.2, 133.6, 131.4, 130.4, 129.0, 78.2 (J_{C-F} = 230 Hz), 71.0, 61.0, 59.0, 58.1, 47.8, 43.7, 39.0, 30.2, 29.1, 15.7, 14.11 (J_{C-F} = 10 Hz), 14.07 (J_{C-F} = 10 Hz).

(*2S,4R*)-1-((*R*)-2-acetamido-3-((6-aminohexyl)thio)-3-methylbutanoyl)-4-hydroxy-*N*-(4-(4-methylthiazol-5-yl)benzyl)pyrrolidine-2-carboxamide (5)

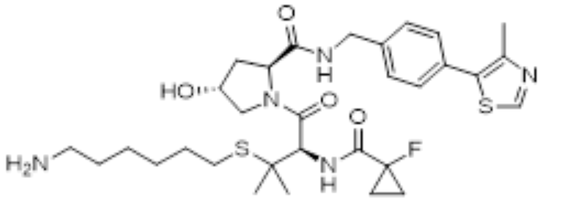

5

Under nitrogen and at 0 °C, a solution of compound **4** (24 mg, 0.045 mmol) in DMF (0.5 mL) was treated with DBU (7.5 µL, 0.049 mmol) followed by *N*-(4-Bromohexyl)phthalimide (15.2 mg, 0.049 mmol). After three hours LCMS indicated the reaction was complete, the reaction mixture was diluted with citric acid solution and extracted with DCM and the volatiles were removed under reduced pressure to afford the crude product. The crude alkylated product was then dissolved in ethanol (2 mL) and treated with hydrazine monohydrate (22 µL, 0.29 mmol) at 70 °C for two hours. The reaction mixture was cooled to room temperature, filtered and purified by preparative HPLC to give the expected amine **5** (17 mg, 60% yield). MS analysis: $C_{31}H_{44}FN_5O_4S_2$ expected 633.3, found 634.5 [M+H$^+$].

^{1}H NMR (500 MHz, MeOD) d 8.88 (s, 1H), 8.55 (s, 1H), 7.47 (d, J=8.5 Hz, 2H), 7.43 (d, J=8.5 Hz, 2H), 4.92 (s, 1H), 4.61 (t, J=8.2 Hz, 1H), 4.56 (d, J=15.4 Hz, 1H), 4.51 (s, 1H), 4.36 (d, J=15.7 Hz, 1H), 3.91 - 3.85 (m, 2H), 2.86 (t, J=7.7 Hz, 2H), 2.59 (t, J=7.2 Hz, 2H), 2.48 (s, 3H), 2.26 (dd, J=8.5, 12.6 Hz, 1H), 2.14 - 2.06 (m, 1H), 1.62 - 1.54 (m, 2H), 1.53 - 1.46 (m, 2H), 1.42 (s, 6H), 1.40 - 1.27 (m, 8H).

^{13}C-NMR (101 MHz, CDCl3, 25 °C) δ: 174.1, 171.4 (J_{C-F} = 20 Hz), 170.7, 152.9, 149.1, 140.2, 133.4, 131.6, 130.4, 128.9, 78.2 (J_{C-F} = 230 Hz), 71.0, 61.1, 58.1, 57.2, 49.9, 43.6, 40.7, 39.1, 30.4, 29.5, 29.2, 28.7, 27.2, 27.1, 25.4, 15.8, 14.0 (J_{C-F} = 10 Hz).

(2S,4R)-1-((R)-3-((6-(2-((S)-4-(4-chlorophenyl)-2,3,9-trimethyl-6H-thieno[3,2-f][1,2,4]triazolo[4,3-a][1,4]diazepin-6-yl)acetamido)hexyl)thio)-2-(1-fluorocyclopropane-1-carboxamido)-3-methylbutanoyl)-4-hydroxy-N-(4-(4-methylthiazol-5-yl)benzyl)pyrrolidine-2-carboxamide (1, AT7)

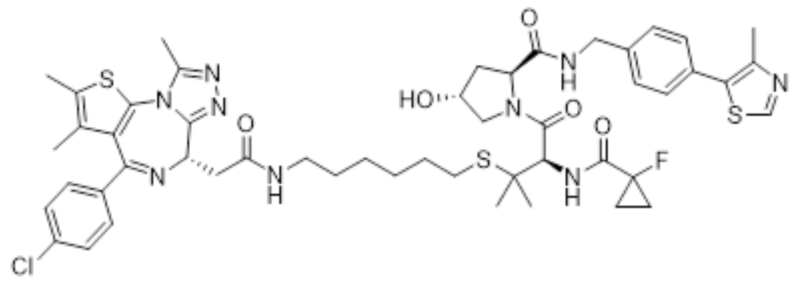

1, AT7

Compound **5** (17 mg, 0.0269 mmol) was dissolved in DMF (0.25 mL) and added to a solution of(*S*)-2-(4-(4-chlorophenyl)-2,3,9-trimethyl-6H-thieno[3,2-f][1,2,4]triazolo[4,3-a][1,4]diazepin-6-yl)acetic acid (+)-JQ1-COOH (11 mg, 0.0269 mmol), COMU (12 mg, 0.0269 mmol), and DIPEA (10 µl, 0.0537 mmol) in DMF (0.25 mL). After stirring at room temperature for 1 h. The crude mixture was dissolved in MeOH,

filtered and purified by preparative HPLC to afford the title compound. Obtained 14.6 mg, 53% yield. MS analysis: $C_{50}H_{59}ClFN_9O_5S_3$ expected 1015.35, found 1016.36 [M+H$^+$].

^{1}H NMR (500 MHz, MeOD) d 8.94 (s, 1H), 8.62 (t, J=5.7 Hz, 1H), 7.74 (d, J=9.1 Hz, 1H), 7.49 - 7.42 (m, 9H), 4.94 (d, J=8.5 Hz, 1H), 4.68 - 4.62 (m, 2H), 4.58 (dd, J=10.5, 10.5 Hz, 1H), 4.52 (s, 1H), 4.39 (dd, J=4.9, 15.6 Hz, 1H), 3.94 - 3.87 (m, 2H), 3.44 (dd, J=9.2, 14.8 Hz, 1H), 3.31 - 3.25 (m, 2H), 3.25 - 3.16 (m, 1H), 2.73 (s, 3H), 2.64 - 2.56 (m, 2H), 2.50 (s, 3H), 2.47 (s, 3H), 2.29 (dd, J=7.7, 13.3 Hz, 1H), 2.17 - 2.09 (m, 1H), 1.72 (s, 3H), 1.57 - 1.47 (m, 4H), 1.44 (s, 6H), 1.41 - 1.32 (m, 8H).

^{13}C-NMR (101 MHz, CDCl3, 25 °C) δ: 174.1, 172.6, 171.5 ($J_{C–F}$ = 20 Hz), 170.9, 166.3, 157.0, 153.0, 152.2, 148.8, 140.3, 138.1, 138.0, 133.6, 133.5, 133.4, 132.1, 132.0, 131.4, 131.3, 130.4, 129.8, 128.9, 78.2 ($J_{C–F}$ = 230 Hz), 71.0, 61.1, 58.1, 57.3, 55.2, 49.9, 43.6, 40.4, 39.0, 38.7, 30.6, 30.3, 29.8, 29.3, 27.6, 27.1, 25.6, 15.8, 14.4, 14.1 ($J_{C–F}$ = 10 Hz), 14.0 ($J_{C–F}$ = 10 Hz), 12.9, 11.6.
